# Nutritive Value and Cannabinoid Potency of Diverse Hemp (*Cannabis sativa* L.) Varieties Grown under Different Light Conditions and Harvested across Multiple Time Points as a Possible Feed Source for Livestock

**DOI:** 10.1021/acs.jafc.5c16989

**Published:** 2026-04-30

**Authors:** Jennifer M. Duringer, Ashley Saindon, Agung Irawan, Serkan Ates, Korey J. Brownstein, Kelly M. Gude, Chris Ringo, Chad Alan Kinney, Mark Berhow, Jeffrey Steiner

**Affiliations:** † Department of Environmental & Molecular Toxicology, 2694 Oregon State University, 139 Oak Creek Building, Corvallis, Oregon 97333, United States; ‡ Department of Animal and Rangeland Sciences, 2694 Oregon State University, Corvallis, Oregon 97331, United States; § 17123 USDA, ARS, NCAUR, Functional Foods Research Unit, Peoria, Illinois 61604, United States; ∥ Global Hemp Innovation Center, 2694 Oregon State University, Corvallis, Oregon 97331, United States; ⊥ Institute for Cannabinoid Research, Colorado State University, Pueblo, Colorado 81001, United States

**Keywords:** chemical composition, feed ingredient, industrial hemp, livestock, nutrition

## Abstract

Effects of two nighttime light interruption conditions and three inflorescence harvest times were studied on two photoperiod-insensitive and three photoperiod-sensitive varieties of industrial hemp (*Cannabis sativa* L.) grown in California, USA, to determine how these affected agronomic and nutritive values relevant to animal feed metrics, as well as the expression of cannabinoids. Cannabinoid potency was quantified using a newly developed UPLC-DAD method. Light sensitivity, light intensity, and harvest time significantly influenced agronomic and nutritional characteristics, in addition to the plant’s cannabinoid profile. High light intensity increased agronomic and nutritional variables, but the response varied by genotype. Cannabinoids increased in photosensitive varieties grown under high light intensity, while the latter harvests demonstrated the highest values in all varieties. This indicates that specific plant genotypes and their response to light availability may be a means to optimize hemp’s nutritional profile and address safety concerns surrounding cannabinoid expression for use as a livestock feed source.

## Graphical abstract

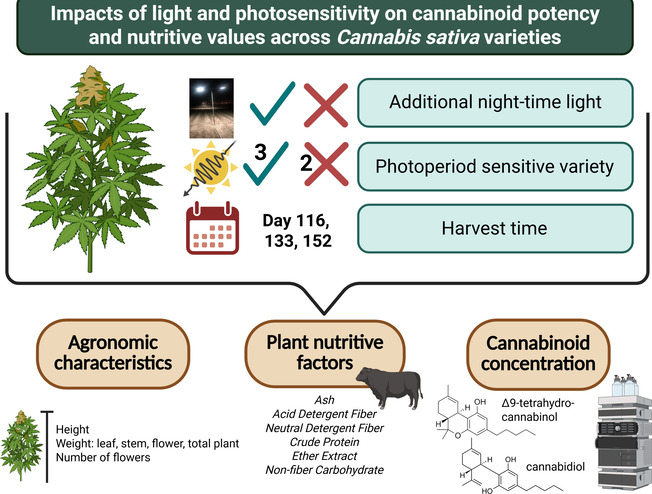

## 1 Introduction

The 2018 Farm Bill removed industrial hemp (*Cannabis sativa* L.) from the Controlled Substances Act, thereby making hemp an agricultural commodity.^1^ Hemp has a wide array of uses, the most well-known of which are grain for nutrition, fiber for textiles, and flower for cannabinoids and other classes of phytocompounds.^2^ Currently, most states in the United States have implemented programs for regulating industrial hemp cultivation and the testing of cannabinoids.

Phytocannabinoids are a group of structurally similar compounds described as terpenophenolic that are primarily found in *C. sativa* ^3^ (Figure 1). Cannabinoids are produced and stored in glandular trichomes located mainly in female flowers.^4^ In resin-type cultivars, total cannabinoids are generally present at concentrations of 16–20% in the flower, 1–2% in the leaf, and are not detectable in the stem or roots.^5^ Cannabinoids are also not present in the seeds (grain) in planta.^6^ Any cannabinoids detected on seeds are likely due to contamination from the dust of floral plant structures during harvest and processing. There is general regulatory concern about cannabinoids entering the human food chain, so accurate testing is necessary to detect cannabinoids in Hempseed cake, herbage, or post-extracted biomass fed to livestock and poultry, while also following any residues through metabolism and production of animal food products.

**Figure 1:**
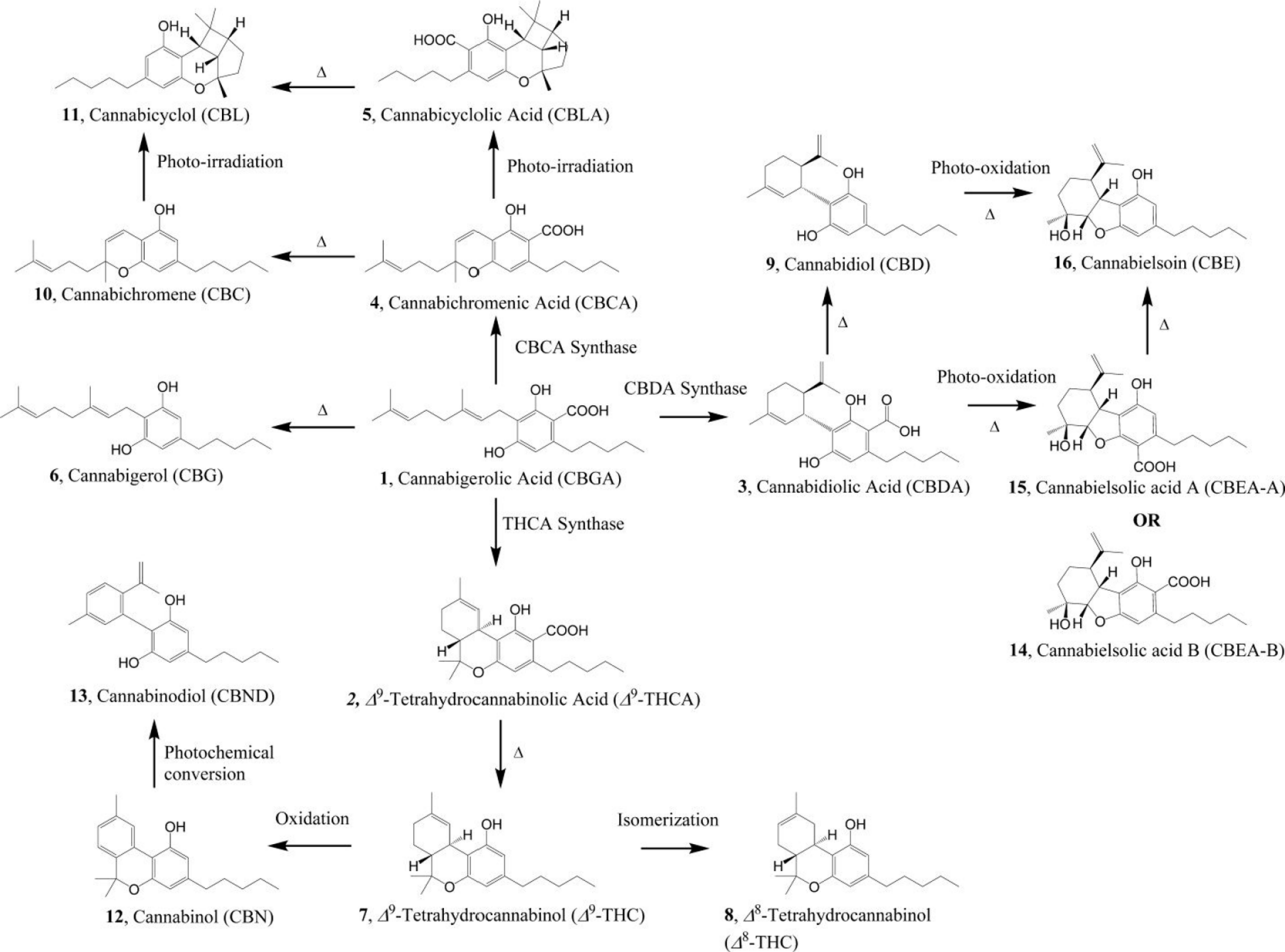
Cannabinoid biosynthesis pathway as described by Lewis et al. (2017). (The delta symbol indicates a thermal reaction.)

Cannabidiol (CBD) is often extracted from flowers and leaves using an ethanol-based procedure to concentrate and purify the oil. After the CBD is removed, large quantities of high-quality post-extract (spent) biomass remain with no approved standard uses or commercial markets for the byproduct. This and other processed hemp products noted by various commodity groups indicate that hemp is an available, viable option as a feed source for livestock.^7,8^ Spent hemp biomass has a high energy content, as seen by the high net energy for lactation and total digestible nutrient values^9^ (Table 1). The amount of protein in spent hemp biomass is equivalent to that of alfalfa, with substantially lower neutral detergent fiber (NDF) content. Furthermore, the mineral content of spent hemp biomass, in particular zinc, is superior to that of alfalfa. All the above nutritive parameters indicate the potential for spent hemp biomass to be an excellent substitute or supplement to alfalfa or other high-energy components in livestock feed rations.^9^ However, there are regulatory concerns about the safety and nutritional considerations of feeding whole hemp plants and/or spent hemp biomass to livestock, as well as utilizing ensiled or pelleted herbage or Hempseed cake, specifically regarding the potential for deposition of cannabinoid residues in food-producing animals.^10–13^

**Table 1:** Nutritive Analysis of Spent Hemp Biomass^a^

**parameter**	**hemp**	**alfalfa**
dry matter (%)	89.6	88.6
crude protein	19.6	21.8
neutral detergent fiber (%)	22.4	37.9
acid detergent fiber (%)	18.7	31.6
net energy for lactation (Mcal/kg)	1.76	1.39
total digestible nutrients (%)	76.8	60.0
Ca (%)	2.46	1.52
K (%)	2.26	1.96
P (%)	0.77	0.22
Mg (%)	0.81	0.38
Na (%)	0.06	0.09
S (%)	0.33	0.34
Cu (ppm)	19.0	11.0
Fe (ppm)	321	258
Mn (ppm)	169	42
Mo (ppm)	<0.3	<0.1
Zn (ppm)	116	17
Se (ppm)	0.42	0.43
relative feed value	309	158

a Analysis was performed by a commercial laboratory (SDK Laboratories, Hutchinson, KS).

Hemp farmers are interested in marketing hemp for utilization by livestock, as they would like to recover revenue from the extracted hemp oil market and alleviate processing bottlenecks in the supply chain.^14–16^ A survey in the United States determined that consumers overwhelmingly support feeding hemp to livestock.^17^ Either as a post-extracted spent biomass byproduct or as a whole plant, industrial hemp would also give livestock managers an additional option for a feed/forage source as they develop feeding regimens for their animals, providing resilience when feed markets fluctuate^18,19^ or undergo significant shortages due to weather, contamination, and other issues.

Currently, the most significant concern when considering feeding hemp biomass to livestock is its cannabinoid content, specifically the amount of the psychoactive cannabinoid delta-9-tetrahydrocannabinol (Δ^9^-THC).^20,21^ Current federal law specifies that Δ^9^-THC concentrations cannot exceed 0.3% of the dry weight in plants grown as industrial hemp. Noncompliant hemp is required by law to be immediately destroyed on-site, representing a financial loss to growers.^22^ There are concerns by regulatory agencies that Δ^9^-THC could cause biological effects in animals or be accumulated in resultant animal food products destined for human consumption, including milk and meat.^19,23^

To prepare for approval of spent hemp biomass as a feed supplement, there is a critical need to assess both the nutritive quality and the concentrations of the major cannabinoids, including Δ^9^-THC, CBD, and their acids, in plant materials from different hemp varieties to better understand the effects of plant-dependent developmental variables on these factors, including time of harvest. By doing so, research surrounding the establishment of dynamic livestock feeding programs could be conducted with hemp, using animal and human safety as the primary drivers in targeted metabolic studies. The primary goal of this study was to quantify cannabinoids in five hemp varieties with variable growing conditions using a high-throughput analytical method, in addition to characterizing the biomass production and nutritive values as indices of importance to livestock producers considering *Cannabis* as a herbage resource.

## 2 Materials and Methods

### 2.1 Field Preparation, Cultivation, and Harvest

The field production location was at the Imperial Valley Conservation Research Center, Brawley, CA, USA (32.955 N, 115.557 W), which is located at approximately 70 m below sea level on soil described as well-draining silty clay.^24^ Irrigation water was provided via a gravity surface canal, the source of which is the Colorado River.^25^ This is a particularly warm and arid region, with an average annual maximal temperature of around 31 °C and an average annual rainfall of only 6.63 cm. Over the course of the experiment, daily average temperatures were between 9 and 26 °C, with an average daily rainfall of 0.56 cm, which is slightly drier than normal.^26^ Additional climate details can be found in Supplementary Table 1.

Based on the initial soil test and pH of 8.2, a preplant fertilizer application was incorporated at rates of 27 kg ha^–1^ nitrogen; 32 kg ha^–1^ P_2_O_5_; 0.5 kg ha^–1^ boron; 2 kg ha^–1^ zinc; and 2 kg ha^–1^ copper. After fertilizer incorporation by discing, 181 kg ha^–1^ of fine gypsum and 4.5 kg ha^–1^ of anticrusting polyacrylamide (PAM) compound (Silt Bond) were spread on the field and incorporated into the top ∼1 cm of soil before listing the planting beds. After listing and shaping planting beds on 102 cm centers, drip tape was buried 6.0–7.5 cm deep and 10 cm off the center of the bed. The drip tape had laser-drilled, 0.4 cm low-flow emitter holes that were spaced 30 cm apart.

#### 2.1.1 Planting by Direct Seeding

On December 18, 2019, five varieties (designated E for entry, Supplementary Table 2) of industrial hemp were planted using a four-row vacuum-type precision planter mounted on tractor tool bars. Each mounted planter box was placed on-center immediately following 1.02 m bed shapers that formed four planting beds at a time. Seeds of each variety were planted 6.4 mm deep, 15 cm apart, into the top middle of the shaped beds. Each plot was four beds wide and was approximately 29 m long. Each of the five varieties was replicated four times and arranged in a randomized complete block design with five ranges 29 m long across the length of a 154 m field (Supplementary Figure 1).

After planting, water was applied on 23 December through movable line sprinklers until seedling emergence and plants were established, which took approximately 4 weeks. Sprinkler water applications were done as frequently as twice per week, with the aim of not letting the soil surface dry and crusting while the seedlings emerged. Ninety-five percent of the stand establishment was achieved by 10 January. After the stand was established, E1 and E2 (photoperiod-insensitive varieties) were hand-thinned by a commercial crew to 30 cm spacings, while E3, E4, and E5 (photoperiod-sensitive varieties) were thinned to 45 cm spacings in the rows.

For nutrient management during the growing season, leaf petiole samples from E3 were collected by a commercial agricultural consulting company from apical plant growing points every 2 weeks. Tissue samples were sent to a commercial testing laboratory for analysis. There were no guidelines for hemp fertilizer applications at this time, but standard crop nutrient status charts indicated whether a wide range of inorganic nutrients were deficient, low, adequate, high, or very high. When plant petiole samples tested low for nutrients, a custom liquid fertilizer mixture for the needed nutrients was applied by fertigation through the drip irrigation delivery system based on the recommendation of the commercial consultant.

#### 2.1.2 Cultivation

The last sprinkler irrigation was timed to moisten the soil surface before cultivation. Established seedling stands were mechanically cultivated using drop-down knives that protected the seedlings within 3.8 cm of both sides of the planting rows. Shallow banana knives cultivated the shoulder of the beds, which were 7.6 cm deep. One day later, knives loosened soil on the shoulder and furrow; shovels then formed the furrow and reshaped the shoulders of the raised beds. Excellent weed control was achieved by mechanical cultivation without herbicides or the use of plastic culture. Manual weeding was performed as needed.

#### 2.1.3 Soil Moisture Monitoring for Irrigation Timing

Three Watermark electronic soil moisture sensors were placed at a depth of 20 cm in the planting row of the E3 sentinel variety to give continuous soil moisture tension readings. The sensor readings were processed in a controller and relayed by a commercial service to a database through a wireless phone network to a website. The target point for water applications was approximately 70 kPa of soil water tension, at which point water was applied through the buried drip tape system. Based on experience, we initially did two water applications through the drip systems per week while seedling roots became established to avoid moisture stress due to the initial limited rooting depth. Once the plants were established, water applications were made when the soil moisture tension approached or exceeded 70 kPa. Water was applied at a rate of 360 L/h for 3–4 h per application, typically once or twice per week, depending upon temperature and whether precipitation had occurred.

#### 2.1.4 Night Period Light Interruption Treatments

Solar light fixtures mounted onto poles 3.1 m tall were installed 15.2 m apart between the middle two of the four rows of each plot (Supplementary Figure 1). Lights were timed to turn on and interrupt the night dark period for 2 h between 00:00 and 02:00 for 64 days from January 4 to March 8, 2020. Each light fixture contained two nominally 7000 lm LED bulbs (Tecessory SolarMax 120W = 240 LED Solar Powered Street Light w/Remote Control (Fountain Valley, CA, USA)). The bulbs were attached to the pole via perpendicular arms to the planted rows, approximately 2.1 m wide, and held 3 m above the planted bed surface. The poles were installed 15 m apart in the furrow between rows eight and nine of the 16-row wide experimental area.

Plants proximal to light fixtures were designated as exposed to “high” interrupted light conditions, while those that were halfway between two light fixtures were considered to be exposed to “low” interrupted light conditions. The high-light plants were found to be consistently exposed to 50–100 lx during the interrupted night period in addition to normal daytime sunlight exposure, while low-light plants were consistently exposed to <30 lx during the interrupted night period in addition to normal daytime sunlight exposure.

#### 2.1.5 Harvest Timing

Whole plants were sampled during three periods of the spring. Early -season sampling was done approximately at days 116 after seeding, midseason on day 133, and late-season on day 152. At each harvest time, two whole plants, including roots, in each block for each of the five varieties, were sampled at random within 2 m of the proximal ends of plots to the lights and distally to the light within a 2 m length of the middle of the range (7.25 m from the lights).

### 2.2 Plant Sample Preparation

Sampled plants were separated into roots, stem, leaf, and flower (inflorescence) parts, placed in paper bags, then dried at ambient temperature to <10% water content in an outdoor barn with fans circulating air at ambient temperature/humidity in early summer (very dry conditions). After drying, all plant part components were weighed, and the leaf-to-stem ratio (LSR) was calculated by dividing the dry weight of the leaf portion by the dry weight of the stem portion of the plants. Plant samples were roughly ground in a grain mill before being shipped to Oregon State University (Corvallis, OR, USA).

To standardize ground particle size, plant grinding was done with either a CT 293 Cyclotec Laboratory Mill or a Tecator Cyclotec 1093 Sample Mill (Foss, Denmark). Flowers were ground through a 1 mm screen for cannabinoid quantification. Stems and leaves were ground with a 2 mm screen for corresponding nutritional analyses. As two plants were sampled from each specific collection site, the roughly ground flowers of both plants from the same site were randomly subsampled and mixed together. Thus, after processing in the sample mill, each variety of hemp was represented by eight samples per harvest, with four samples generated from each light condition. With five varieties of hemp sampled over three harvest periods, a total of 120 hemp samples were generated.

### 2.3 Plant Nutritive Value Measurements

Ground flower, stem, and leaf samples were analyzed for their nutritive value using methods established by AOAC International^27^ for dry matter (DM) (930.15), ash (942.05), and ether extract (920.39) (EE). All nutritive value parameters are expressed as a percentage of DM. The crude protein (CP; 6.25 × N) concentration of samples was determined by an LECO FP828 (Leco Corporation, St. Joseph, MI, USA). NDF and acid detergent fiber (ADF) were analyzed sequentially using an ANKOM 200 Fiber Analyzer (ANKOM Technology Corp., Macedon, NY, USA).^28,29^ NDF was analyzed with the inclusion of heat-stable α-amylase and sodium sulfite. nonfiber carbohydrate (NFC) concentration was calculated using a modified equation: NFC = 100 – ([%NDF – 2] + CP + Ash + EE), which assumes an estimate of the neutral detergent insoluble CP concentration of 2.0%.^28^

### 2.4 Inflorescence Cannabinoid Extraction

The single-step extraction method described builds upon procedures developed previously for the quantification of cannabinoids in hemp.^30,31^ Briefly, 0.100 g (±0.005 g) of ground flower was weighed in triplicate into glass scintillation vials. Next, 20 mL of absolute ethanol was added to each sample, which was then placed into a sonicator (Branson 8800 Ultrasonic Cleaner, Branson Ultrasonics, Danbury, CT, USA) for 30 min. Preliminary evaluation compared sonication and Soxhlet extraction protocols and assessed solvent extraction efficiency to cannabinoid recovery and extraction yield. The various solvents assessed included 100% acetonitrile, 100% methanol, 80% methanol/20% 18 MΩ water, 50% DMSO/50% methanol, and 100% acetonitrile/0.1% formic acid. Additionally, we performed a 20-cycle Soxhlet ethanolic extraction of these samples on a BÜCHI (Labortechnik AG, Flawil, Switzerland) Universal Extractor E-800 connected to a BÜCHI Recirculating Chiller F-314, using 1 g of the same sample with 180 mL of ethanol, which was brought to 200 mL after cooling. The samples subjected to the exhaustive Soxhlet extraction were not sonicated. Following sonication, samples rested, protected from light, for 18–24 h at ambient temperature for settling. Next, samples were shaken and allowed to settle, and then 1.5 mL of supernatant was collected into a 3 mL syringe and filtered (25 mm, 0.45 μm PTFE, VWR, Radnor, PA, USA) into an HPLC vial and then subsequently sealed for cannabinoid analysis.

### 2.5 Ultra High-Performance Liquid Chromatography (UPLC) Method Validation

The extraction efficiency of 100% absolute ethanol, 100% methanol, 100% acetonitrile, 80% methanol/20% 18 MΩ water (v/v), 50% DMSO/50% methanol (v/v), 100% acetonitrile/0.10% formic acid, and exhaustive Soxhlet extraction revealed that absolute ethanol extracted the highest percent total of cannabinoids (16.13%), as well as CBD (12.65%) and Δ^9^-THC (0.45%) (Supplementary Tables 3–5). For this initial evaluation, approximately 0.100 g of ground hemp flowers from a single sample was extracted three times sequentially with 10 mL of solvent using sonication for 30 min and allowed to stand at room temperature for over 18 h. All evaluations were done in triplicate. Each extraction was evaluated for its cannabinoid content. The first two extractions yielded over 99% of the extractable cannabinoids, with the third extraction recovering only an additional 0.1–0.5% of the total cannabinoids (Supplementary Table 3). Samples extracted with 100% absolute ethanol using an orbital shaker for 18 h at room temperature yielded lower levels of recoverable cannabinoids (13.56%) compared to using the water bath sonicator (16.13%). Similar results have been found in other raw product extractions.^32,33^

To provide additional evidence for the optimal extraction efficiency of 100% absolute ethanol (the final solvent chosen for cannabinoid quantitation of the field study samples), we performed a 20-cycle ethanolic extraction using an automated Soxhlet extractor system and compared this to a second single-step ethanolic extraction. Both the 20-cycle and single-step methods extracted the same percent total THC, i.e., 0.24% (Supplementary Figure 2a). However, the single-step method extracted a higher percentage of total cannabinoids (Supplementary Figure 2b), as well as more total CBD and CBG (Supplementary Figure 2c,d) in comparison to the 20-cycle method. The single-step extraction method had 7.52% total CBD, while the Soxhlet extraction method had 7.41% total CBD (Supplementary Figure 2c). There was a reduction in the amount of CBDA in the 20-cycle extract. The Soxhlet extraction method required the repetitive boiling of solvents, which may explain why there was less CBDA and more CBD (Supplementary Figure 2e,f) in the 20-cycle extract in comparison to the single-step extracts. Upon heating, the acid forms of cannabinoids are known to decarboxylate to neutral (nonacidic) forms.

### 2.6 Inflorescence Cannabinoid UPLC-Diode Array Detection (DAD) of 16 Cannabinoids

Stock standard solutions of eight cannabinoid acids were prepared in acetonitrile, while the eight decarboxylated cannabinoids were prepared in methanol. The standard stock solution concentration was 100 μg mL^–1^. All the standard solutions were flushed with nitrogen and stored at −80 °C for up to 2 months. For the analyses, water (18 MΩ) was obtained from an Elga Ultra PureLab (Cary, NC, USA) or Milli-Q (Millipore, Bedford, MA, USA) water purification system. HPLC grade of methanol, acetonitrile, formic acid, and dimethyl sulfoxide (DMSO) was purchased from EMD Millipore Corporation (Burlington, MA, USA), Fisher Scientific (Waltham, MA, USA), Honeywell (Muskegon, MI, USA), or JT Baker (Phillipsburg, NJ, USA), while absolute ethanol was obtained from Koptec (King of Prussia, PA, USA). The chemicals ammonium formate and standards for all cannabinoids were purchased from commercial sources (Sigma-Aldrich (St. Louis, MO, USA) and Cerilliant (Round Rock, TX, USA), respectively), specifically: Δ^9^-THC, CBD, cannabigerol (CBG), cannabivarin (CBDV), tetrahydrocannabivarin (THCV), cannabinol (CBN), cannabichromene (CBC), and delta-8-tetrahydrocannibinol (Δ^8^-THC) at 0.5 mg/mL in methanol; and delta-9-tetrahydrocannibinolic acid (THCA), cannabidiolic acid (CBDA), cannabigerolic acid (CBGA), cannabivarinic acid (CBDVA), tetrahydrocannabivarinic acid (THCVA), cannabinolic acid (CBNA), cannabichromene acid (CBCA), and cannabicyclolic acid (CBLA) at 0.5 mg/mL in acetonitrile. Standards of cannabigerovarinic acid (CBGVA), cannabichromevarinic acid (CBCVA), and cannabicyclol (CBL) were 1.0 mg/mL in acetonitrile. Cannabivarin (CBV) solid was purchased from Cayman Chemical (Ann Arbor, MI, USA).

The concentrations of seven cannabinoids and their associated acids, as well as two additional cannabinoids, were analyzed in the inflorescences generated from the field study here, namely, Δ^9^-THC, CBD, CBG, CBDV, THCV, CBN, CBC, Δ^8^-THC, THCA, CBDA, CBGA, CBDVA, THCVA, CBNA, CBCA, and CBLA. UPLC analysis was performed using an Agilent 1290 Infinity System (Santa Clara, CA, USA), controlled by MassHunter software using an Agilent InfinityLab Poroshell 120 EC-C18 column (2.1 × 100 mm, 2.7 μm) held at 55 °C. The two mobile phases used were composed of 5 mM aqueous ammonium formate + 0.1% formic acid (mobile phase A) and acetonitrile + 0.1% formic acid (mobile phase B), with 2.5 min equilibrating at 60% B between injections and the following gradient elution: 0–2 min, 70% B; 2–8 min, 75% B; 8–9 min, 100% B; 9–10 min, 100% B; 10–10.5 min, 70% B; 10.5–11, 70% B. Sample injection volume was 1 μL and the flow rate was set to 0.55 mL/min. Peaks were quantified using DAD (Agilent 1290 Infinity II DAD, Santa Clara, CA, USA) at 228 nm.^30,31^ Quantification was performed using the QQQ Quantitative Analysis program of MassHunter software. Samples were quantified for all 16 cannabinoids against standard curves (0.78–100 μg/mL for all except CBD, which was 25–500 μg/mL) made using commercially available standards.

### 2.7 UPLC-DAD Analysis of 20 Cannabinoids

Further UPLC-DAD analysis of 20 cannabinoids (CBDVA, CBGVA, CBDV, CBV, CBDA, CBGA, THCV, CBG, CBD, THCVA, CBCVA, CBN, CBNA, Δ^9^-THC, Δ^8^-THC, THCA, CBL, CBC, CBCA, and CBLA) was performed on a Thermo Scientific Dionex UltiMate 3000 (Waltham, MA, USA) UPLC system for the NIST CannaQAP lab comparison exercise (Supplementary Figure 3). This faster method allowed for higher sample throughput and determination of analytical robustness across laboratories. Mobile phases A and B consisted of the same solvents as described above; however, the UPLC gradient program was modified to separate each cannabinoid within an 8 min data collection time. The autosampler chamber and column temperatures were 8 and 50 °C, respectively, and the cannabinoids were measured at 228 nm. One μL of sample was injected and separated through a GL Sciences Inc. (Tokyo, Japan) InertSustain C18, 3 μm, 2.1 × 100 mm column at a flow rate of 0.60 mL/min. We applied the following gradient parameters: 0–2 min, 70% B (concave curve 9); 2–5.5 min, 75% B (concave curve 9); 5.5–6 min, 100% B (linear curve 5); 6–7 min, 100% B; 7–7.5 min, 65% B (linear curve 5); 7.5–8 min, 65% B followed by 2.0 min of equilibration at 65% B between injections. The total run time for the instrument was 10 min. Peak areas (mAU/min) were detected and quantified in Chromeleon 7 (v7.2) using the Cobra algorithm. “Auto range” was checked under the baseline noise range, and the Cobra smoothing width was set to “auto” [min]. Inhibit integration was “on” from 0 to 1 min, then set to “off” from 1 to 6.75 min, and turned back “on” at 6.75 min. At 1 min, the minimum area was set to “auto.”

### 2.8 Determining Total Cannabinoid Content

The equation for obtaining the cannabinoid content of hemp was: Cannabinoid analyte (μg/mg) = *C*_e_ × *V*_f_/*W*_s_ (1), where *C*_e_ = concentration of the sample in the extraction solution (μg/μL), *V*_f_ = final volume of the sample (μL), and *W*_s_ = weight of the sample (mg). Cannabinoids are often found in two distinct forms: an acid precursor form and a neutral derivative. These compounds are chemical analogs and are capable of changing structure to transform into the other, most often in the direction of acid to neutral. Calculating the total [cannabinoid] concentration takes into account the potency of both the acid and the neutral form. This can be accomplished by multiplying the concentration of the acid form by 0.877 and then adding that value to the concentration of the neutral form (Code of Federal Regulations 7 CFR 990.1). This concept is displayed in the following formula: Total cannabinoid = ([acidic form] × 0.877) + [neutral form].

### 2.9 Method Validation

Method validation was performed according to the guidelines set by the International Union of Pure and Applied Chemistry^34^ and CODEX.^35^ The method was validated for selectivity, linearity, sensitivity, and precision. The method was deemed selective since it was able to detect each of the compounds within an applicable calibration range. Linearity was calculated by establishing external calibration curves using working solutions containing the cannabinoids. The calibration curves were made by incorporating different concentrations of the mixed standard solution and the corresponding values of the peak area. The concentration of the mixed standard solution was injected four times, and then the regression parameters were calculated. Correlation coefficients (*R* ^2^) better than 0.99 for the tested compounds were obtained (THCA is the exception at 0.98). These results suggested that an external standard calibration could be applied for quantitative purposes. By determining the limits of detection (LOD) and the limits of quantification (LOQ), the sensitivity of the developed method was validated, using the following equations^36^: LOD = 3.3 × σ/*S*, LOQ = 10 × σ/*S* (σ, standard deviation; *S*, slope). The value range of LOD was 0.001–0.004 μg/mg, and that of the LOQ was 0.003–0.023 μg/mg for the 17 different cannabinoids (Supplementary Table 6). Precision was determined by measuring the intraday percentage of the relative standard deviation (%RSD). For intraday precision, four replicates of the mixed standard solutions (100, 50, 25, 10, and 5 μg/mL) were analyzed within 1 day. The overall %RSD for the intraday was 5% for most components, except for CBDA and CBCA, which were calculated at 7% (Supplementary Table 6). Uncertainty for each cannabinoid at each concentration level can be calculated using the formula *U* = *k* × RSD provided by the FDA Office of Regulatory Affairs.^37^ The %RSD used in this calculation was the value generated from the intraday validation. With a 95% confidence level, a coverage factor of *k* = 2.144 for *N* = 15 was used.^38^

### 2.10 Statistical Analyses

The experimental treatments were assigned to plots with the interrupted nighttime light interruption level (*L*) nested in blocks (*B*) and varieties (*V*) randomized within confounded block-light level combinations, and harvest dates (*H*) randomized within *V*. The analysis of variance (ANOVA) was done as a split-plot-type randomized complete block design with the hierarchy: *L* confounded in *B* > *V* > *H*. The model for this design was: *y_ijkl_* = *m* + *B_i_* + *d*_(*i*)_ + *L_j_* + *h*_(*j*)_ + *BL*_*ij*_ + *V_k_* + *BV_ik_* + *LV_jk_* + *BLV_ijk_* + *H_l_* + *BH_il_* + *LH_jl_* + *BLH_ijl_* + *VH_kl_* + *BVH_ikl_* + *LVH_jkl_* + *BLVH_ijkl_* + *e*_(*ij*)*kl*_ where *y_ijkl_* = variable to be measured for the *i*th measured response of the *l*th *H* from the *k*th *V* of the *j*th *L* in the *i*th *B*; *m* is the overall mean; and the four-way interaction for BLVH is used as the error for testing the main effects of Varieties and Harvest Dates and all their interactions. There is no test for the main effect of L because it is confounded with B, as there is no replication of the light within each block.

ANOVA was used to test for treatment effects on agronomic characteristics, nutritive values, and cannabinoid concentrations due to varieties, harvest times, and interrupted nighttime light conditions period using Genstat 21st Edition (VSN International, Hemel Hempstead, U.K.). Significant differences among treatment means were determined by Fisher’s protected least significant difference; all reported differences are *P* ≤ 0.05, unless otherwise indicated. Cannabinoid quantitation statistics, including the development of standard curves, averages, and standard deviations for method validation, were performed in Microsoft Excel (Microsoft, Redmond, WA, USA).

## 3 Results

### 3.1 Agronomic Characteristics

The agronomic measurement responses of photoperiod-sensitive and photoperiod-insensitive varieties grown under different nighttime light interruption intensities and harvest times are shown in Table 2. Three-way interactions (*L* × *E* × *H*) were observed for all measured parameters, except for leaf weights and the leaf-to-stem ratios in hemp plants. Overall, increased light exposure led to taller plants (98.7 vs 49.7 cm) and greater total (181 vs 123 g), stem (69.6 vs 24.1 g), and leaf (73 vs 30 g) weights. Although high light intensity increased the number of flowers per plant (88.0 vs 51.3), this came at the expense of flower weight (38.7 vs 69.2 g).

**Table 2:** Agronomic Characteristics of Photoperiod-Sensitive and Photoperiod-Insensitive Hemp Flowers Sampled at 116, 133, and 152 Days under Two Light Conditions

**light** [Table-fn t2fn1]	**entry**	**harvest** [Table-fn t2fn2]	**plant height (cm)**	**plant weight (g)**	**leaf weight (g)**	**stem weight (g)**	**flower weight (g)**	**flower number per plant**	**leaf to stem ratio**
low	E1	H1	35.6_a_	48.6_a_	29.4	6.3_a_	10.4_a_	25.8	5.2
		H2	32.7_a_	73.1_a_	24.3	5.3_a_	35.9_ab_	36.1	8.1
		H3	31.1_a_	69.3_a_	20.3	19.5_a_	37.1_ab_	24.9	2.1
	E2	H1	47.0_ab_	104.8_ab_	132.5	15.8_a_	58.3_b_	49.6	2.0
		H2	69.2_c_	172.5_bc_	77.9	34.1_ab_	76.5_bc_	127.1	2.3
		H3	96.5_d_	498.0_f_	111.8	175.5_f_	263.0_f_	158.5	0.4
	E3	H1	42.5_ab_	65.4_a_	94.3	9.5_a_	35.4_ab_	32.1	2.4
		H2	45.4_a_	94.6_ab_	99.6	12.6_a_	55.5_de_	63.0	2.2
		H3	49.5_ab_	139.4_ab_	95.5	15.0_a_	102.0_d_	55.5	1.6
	E4	H1	36.2_a_	64_a_	110.4	6.3_a_	36.1_ab_	28.8	3.5
		H2	34.7_a_	69.2_a_	55.9	6.7_a_	42.8_ab_	25.2	3.1
		H3	36.8_a_	77.3_a_	87.4	8.6_a_	48.0_ab_	26.8	2.3
	E5	H1	62.9_c_	97.0_ab_	124.6	12.9_a_	55.1_b_	43.4	2.3
		H2	59.4_bc_	130.0_ab_	83.6	16.9_a_	76.5_d_	44.1	2.3
		H3	65.4_c_	143.3_abc_	68.3	16.1_a_	102.3_d_	29.3	1.7
high	E1	H1	41.1_a_	72.3_a_	23.0	9.9_a_	29.9_ab_	44.9	3.7
		H2	40.6_a_	58.3_a_	32.0	9.2_a_	28.0_ab_	100.0	2.5
		H3	35.6_a_	54_a_	11.6	5.3_a_	33.0_ab_	27.4	3.0
	E2	H1	85.1_d_	263.9_de_	30.8	79.6_c_	51.8_de_	88.5	1.9
		H2	62.9_bc_	225.5_cd_	61.9	44.5_bc_	103.1_d_	188.5	2.6
		H3	60.0_bc_	187.4_cd_	50.3	15.0_a_	129.5_de_	74.3	2.9
	E3	H1	126.2_ef_	251.2_cd_	20.5	119.5_cd_	28.7_ab_	61.8	0.9
		H2	140.0_f_	247.8_cd_	26.5	126.9_d_	21.3_ab_	202.0	0.9
		H3	114.0_e_	173.0_bc_	16.6	80.9_c_	43.9_bc_	91.3	0.9
	E4	H1	110.2_de_	190.6_cd_	21.6	66.0_bc_	14.3_a_	59.1	2.5
		H2	115.9_e_	143.4_abc_	19.8	61.6_bc_	25.9_ab_	88.8	1.1
		H3	101.3_e_	154.4_bc_	18.6	59.1_bc_	32_ab_	106.4	1.4
	E5	H1	123.2_e_	300.5_e_	29.0	135.5_d_	4.0_a_	22.5	1.2
		H2	169.5_g_	218.3_cd_	36.6	127.3_d_	7.4_a_	73.4	0.7
		H3	161.3_g_	235.8_de_	22.9	118.8_cd_	41.3_ab_	120.8	0.6
**SEM** L × *E* × *H*			9.00	36.4	17.89	13.41	15.18	23.73	1.00
**P** [Table-fn t2fn3] light (*L*)			*	*	*	*	*	*	*
**P** entry (*E*)			*	*	*	*	*	*	*
**P** harvest (*H*)			ns	ns	*	ns	*	*	ns
**P** *L* × *E*			*	*	ns	*	*	ns	ns
**P** *L* × *H*			*	*	*	ns	*	ns	ns
**P** *E* × *H*			ns	ns	ns	*	*	ns	ns
**P** *L* × *E* × *H*			*	*	ns	*	*	ns	ns

1 Light condition: Low = no additional light; High = application of lamp energy.

2 H = harvest time point (H1 = day 116, H2 = day 133, and H3 = day 152).

3 ns = not significant (*P* > 0.05), * = significance (*P* < 0.05).

^a–e^Means within a column with different superscripts differ (*P* < 0.05).

Under low light conditions, plant weights gradually increased with delayed harvest (76 to 109 to 185 g). In contrast, plants grown under high light reached their maximum weight at the earliest harvest stage, maintaining similar or slightly reduced weights as harvest was delayed (203 to 179 to 161 g). The extent of weight reduction over time varied depending on the plant entry. High light significantly increased plant height in E3, E4, and E5 (127.6 vs 42.5, 110.2 vs 36.2, and 116.2 vs 62.9 cm, respectively) at the first harvest time point, with this growth continuing steadily until the second harvest before stabilizing. Interestingly, E1 showed almost no response to increased light (41.1 vs 35.6 cm), while E2’s response was noticeable only up to the first harvest (85.1 vs 47.0 cm) and was less pronounced compared to E3, E4, and E5.

### 3.2 Plant Nutritive Value

Tables 3–5 present the nutritional characteristics of different plant parts from various hemp varieties grown under different light intensities and harvesting regimes. Overall, the flowers and leaves of industrial hemp exhibited high nutritive value with average crude protein (CP) values of 25.5 and 19.4%, respectively. The ether extract (EE) contents of the flowers and leaves were 14.3 and 6.6%, respectively. Regarding the nutritive value of flowers, hemp varieties (*E*), harvest time (*H*), and their interaction (*E* × *H*) exhibited a three-way significant interaction effect on the CP, ADF, NDF, NFC, and EE content of hemp flowers (Table 3), while ash content was only affected by a *L* × *E* interaction effect. Chemical profiles were highly variable among varieties, but E3 and E4 exhibited the highest CP (26.8% each versus 22.1–26.2% for the other varieties) and lower NDF contents (average of 4.0% compared to 26.6–35.9% for the other varieties), while E2 had significantly lower nutritive values as shown by smaller CP and NFC and higher ADF and NDF content. For the harvest time, a markedly higher CP and lower fiber fraction (ADF and NDF) were observed at the first and second harvests versus the third harvest time (28.7–28.9 vs 22.8%), and for the hemp flowers that were exposed to high light intensity versus low light intensity (26.7 vs 24.3%).

**Table 3:** Nutritive Value of Photoperiod-Sensitive and Photoperiod-Insensitive Hemp Flowers Sampled at 116, 133, and 152 Days under Two Light Conditions

			**chemical composition** [Table-fn t3fn3] (**%)**					
****light** [Table-fn t3fn1]**	**entry**	****harvest** [Table-fn t3fn2]**	**ash**	**ADF**	**NDF**	**CP**	**EE**	**NFC**
high	E1	H1	14.2	19.4_c_	28.5_d_	26.5	13.9_bc_	19.0
		H2	21.7	20.5_c_	31.3_d_	26.5	10.8_a_	11.7
		H3	17.8	22.1_c_	31.7_d_	24.7	9.7_a_	18.2
	E2	H1	13.8	19.9_c_	29.3_d_	26.3	9.3_a_	23.4
		H2	16.5	21.3_c_	31.1_d_	23.4	15.1_bc_	16.0
		H3	13.7	26.8_d_	38.2_e_	19.5	17.7_c_	12.9
	E3	H1	13.0	14.5_a_	22.1_bc_	30.7	14.9_c_	21.3
		H2	19.5	12.7_a_	18.3_a_	31.6	11.3_ab_	21.3
		H3	12.7	17.5_b_	26.7_e_	23.2	13.4_ab_	26.0
	E4	H1	14.5	14.4_b_	20.8_ab_	31.2	9.6_a_	25.9
		H2	13.3	16.4_b_	22.9_bc_	29.3	12.5_ab_	24.1
		H3	13.9	16.5_b_	23.8_bc_	23.2	11.2_ab_	29.9
	E5	H1						
		H2	14.2	12.2_a_	17.3_a_	33.9	8.6_a_	30.7
		H3	14.0	16.8_b_	26.8_bc_	23.5	12.6_ab_	25.2
low	E1	H1	15.6	18.9_b_	27.1_c_	26.0	11.9_ab_	21.4
		H2	20.2	21.5_c_	32.5_d_	24.9	9.9_a_	14.5
		H3	17.2	21.7_c_	30.6_d_	24.9	11.1_ab_	18.3
	E2	H1	11.6	25.3_d_	37.5_e_	21.8	18.2_c_	12.9
		H2	12.2	30.9_e_	44.9_f_	19.8	17.0_c_	8.2
		H3	14.5	24.4_d_	34.3_de_	22.0	15.1_bc_	16.2
	E3	H1	13.8	14.7_a_	22_ab_	26.6	18.4_c_	21.3
		H2	20.7	13.9_a_	20.7_ab_	25.4	21.7_d_	13.5
		H3	14.1	20.0_c_	32.5_de_	23.9	18.7_c_	12.8
	E4	H1	13.0	17.3_b_	26.3_c_	27.0	19.4_cd_	16.2
		H2	19.4	18.0_b_	26.5_c_	27.9	16.8_c_	11.5
		H3	13.6	15.2_a_	23.0_bc_	25.7	16.4_c_	23.3
	E5	H1	13.9	20.5_c_	31.3_d_	23.4	19.7_cd_	13.6
		H2	13.4	20.4_c_	30.2_d_	25.0	19.1_cd_	14.3
		H3	13.0	19.2_c_	30.8_d_	21.1	18.3_cd_	18.7
**SEM** *L* × *E* × *H*			1.36	1.21	1.63	1.12	1.57	2.34
**P** [Table-fn t3fn4] light (*L*)			ns	*	*	*	*	*
**P** entry (*E*)			*	*	*	*	*	*
**P** harvest (*H*)			*	*	*	*	ns	*
**P** *L* × *E*			ns	*	*	*	*	*
**P** *L* × *H*			ns	*	*	*	ns	ns
**P** *E* × *H*			*	*	*	*	ns	ns
**P** *L* × *E* × *H*			ns	*	*	ns	*	ns

1 Light condition: Low = no additional light; High = application of lamp energy.

2 *H* = harvest time point (H1 = day 116, H2 = day 133 and H3 = day 152).

3 ADF = acid detergent fiber; NDF = neutral detergent fiber; CP = crude protein; EE = ether extract; NFC = nonfiber carbohydrate.

4 ns= not significant (*P* > 0.05), * = significance (*P* < 0.05).

^a–e^Means within a column with different superscripts differ (*P* < 0.05).

The nutritive value of leaves was similar to the observed results in the flowers, which were affected by *L* × *E* × *H* on all nutrient variables except the ash content (Table 4). High light intensity resulted in higher CP (20.9 vs 17.9%) and NFC (30.7 vs 23.6%) and lower ash contents (23.4 vs 30.1%) of hemp leaves than those that received low light intensity, but this significant effect was only observed in hemp entries under high light intensity for CP. Also, CP content was not different among different harvest times under high inten sity, but a difference was observed under low light intensity (25.0 to 24.6 to 23.5%). A greater effect of harvest time on NDF content was found on hemp plants that received low light intensity than those under high light intensity; NDF content of leaves at second and third harvest times was increased by 19.0–33.4% under low light intensity, while it was increased by 6.0–10.1% under high light intensity. Conversely, NFC content after the second and third harvest times was reduced by 20.6–61.2% in hemp under high-intensity light and 13.1–14.7% on hemp under low-intensity light.

**Table 4:** Nutritive Value of Photoperiod-Sensitive and Photoperiod-Insensitive Hemp Leaves Sampled at 116, 133, and 152 Days under Two Light Conditions

			**chemical composition** [Table-fn t5fn3] (**%)**					
****light** [Table-fn t5fn1]**	**entry**	****harvest** [Table-fn t5fn2]**	**ash**	**ADF**	**NDF**	**CP**	**EE**	**NFC**
high	E1	H1	27.6	14.1_c_	25.8_d_	24.7_ef_	5.5_bc_	18.3_ab_
		H2	27.7	12.7_a_	20.1_bc_	18.6_bc_	8.6_de_	27.0_bc_
		H3	30.6	14.5_d_	23.5_c_	26.3_f_	4.4_ab_	17.2_a_
	E2	H1	22.9	12.9_bc_	19.1_bc_	15.8_ab_	4.0_a_	40.1_f_
		H2	29.3	12.9_bc_	20.1_bc_	16.4_ab_	10.8_n_	25.4_bc_
		H3	27.6	14.3_cd_	20.3_c_		8.7_de_	
	E3	H1	19.7	12.6_bc_	18.1_ab_	20.7_cd_	3.7_a_	39.8_f_
		H2	22.0	13.9_c_	21.3_c_	26.1_f_	5.1_ab_	27.6_cd_
		H3	20.1	11.5_b_	19.8_ab_	20.1_cd_	6.7_cd_	35.3_ef_
	E4	H1	20.8	13.1_c_	19.5_ab_	25.2_ef_	7.6_cd_	28.9_cd_
		H2	21.8	15.1_d_	28.9_e_	16.3_ab_	4.6_ab_	30.5_de_
		H3	21.2	12.9_bc_	21.9_c_	18.9_bc_	6.2_c_	33.8_ef_
	E5	H1	18.9	12.2_bc_	17.7_ab_	18.1_bc_	6.8_cd_	40.5_f_
		H2	20.5	14.0_c_	19.9_bc_	24.9_ef_	4.2_a_	32.5_de_
		H3	20.4	12.8_bc_	21.3_c_	20.6_cd_	7.0_cd_	32.8_def_
low	E1	H1	28.2	11.9_bc_	27.7_e_	16.6_ab_	3.4_a_	26.2_bc_
		H2	31.3	14.7_d_	23.4_cd_	17.5_bc_	8.3_de_	21.4_bc_
		H3	28.9	13.7_cd_	22.4_cd_	15.3_a_	5.0_ab_	30.3_cd_
	E2	H1	29.9	11.0_b_	15.9_a_	22.8_de_	5.7_bc_	27.7_cd_
		H2	28.0	12.9_c_	18.1_a_	14.4_a_	11.2_f_	30.3_cd_
		H3	20.7	16.5_e_	23.1_cd_		8.0_d_	
	E3	H1	31.7	10.8_ab_	16.9_a_	17.7_bc_	4.6_ab_	31.0_cd_
		H2	30.8	11.4_b_	27.3_e_	17.7_bc_	10.5_f_	15.8_a_
		H3	32.6	11.5_b_	18.6_a_	19.4_c_	6.5_bc_	24.8_bc_
	E4	H1	30.3	11.0_bc_	16.6_a_	16.7_ab_	3.8_a_	34.7_de_
		H2	36.1	12.4_bc_	35.2_f_	17.4_ab_	4.7_ab_	8.6_a_
		H3	30.7	10.9_b_	26.7_de_	19.2_c_	6.1_bc_	19.4_ab_
	E5	H1	29.7	9.4_a_	15.2_a_	21.8_cd_	9.9_ef_	25.3_bc_
		H2	32.0	13.2_c_	34.7_f_	14.7_a_	6.7_bc_	13.8_a_
		H3	30.2	11.0_b_	23.2_cd_	19.3_c_	7.7_cd_	21.6_ab_
**SEM** *L* × *E* × *H*			1.36	0.61	1.46	1.48	0.72	2.80
**P** [Table-fn t5fn4] light (*L*)			*	*	*	*	*	*
**P** entry (*E*)			*	*	*	*	*	*
**P** harvest (*H*)			ns	*	*	*	*	*
**P** *L* × *E*			*	*	*	*	*	*
**P** *L* × *H*			ns	*	*	*	*	*
**P** *E* × *H*			ns	*	*	*	*	*
**P** *L* × *E* × *H*			ns	*	*	*	*	*

1 Light condition: Low = no additional light; High = application of lamp energy.

2 *H* = harvest time point (H1 = day 116, H2 = day 133 and H3 = day 152).

3 ADF = acid detergent fiber; NDF = Neutral detergent fiber; CP = crude protein; EE = ether extract; NFC = nonfiber carbohydrate.

4 ns = not significant (*P* > 0.05), * = significance (*P* < 0.05)

^a–f^Means within a column with different superscripts differ (*P* < 0.05).

**Table 5:** Nutritive Value of Photoperiod-Sensitive and Photoperiod-Insensitive Hemp Stems Sampled at 116, 133, and 152 Days under Two Light Conditions

			**chemical composition** [Table-fn t4fn3], **%**					
****light** [Table-fn t4fn1]**	**entry**	****harvest** [Table-fn t4fn2]**	**ash**	**ADF**	**NDF**	**CP**	**EE**	**NFC**
high	E1	H1	8.4	47.2_a_	62.9	8.6	0.5	21.7
		H2	7.4	46.1_a_	61.6	8.1	0.9	24.0
		H3	7.5	48.8_ab_	65.1	8.6	0.7	20.1
	E2	H1	10.8	49.4_ab_	64.3	7.1	0.7	19.1
		H2	6.7	52.0_bc_	67.5	7.0	1.1	19.7
		H3	7.1	54.3_c_	69.0	6.7	0.9	18.3
	E3	H1	8.5	52.3_bc_	67.6		0.5	
		H2	7.4	54.8_c_	70.1	7.0	0.9	16.7
		H3	6.0	56.8_d_	72.8	4.1	1.0	18.1
	E4	H1	9.2	47.4_a_	64.0	8.5	0.8	19.5
		H2	6.3	55.1_cd_	70.6	6.5	0.9	17.6
		H3	6.9	57.0_d_	72.7	4.9	0.7	16.7
	E5	H1	6.7	54.0_cd_	70.7	6.1	0.5	18.1
		H2	7.1	52.9_c_	69.4	6.7	1.3	17.5
		H3	4.6	59.4_e_	74.2	2.7	0.9	19.6
low	E1	H1	11.1	44.9_a_	61.0	8.6	0.7	20.5
		H2	7.5	47.5_a_	64.1	8.9	0.8	20.7
		H3	8.0	50.9_abc_	67.7	7.9	1.1	17.3
	E2	H1	11.4	56.0_de_	71.4	3.8	0.5	14.9
		H2	8.4	50.3_ab_	66.4	7.7	1.0	18.5
		H3	6.2	52.1_bc_	68.2	5.4	0.7	21.5
	E3	H1	10.8	49.9_ab_	64.6	5.3	0.6	20.6
		H2	6.3	49.3_ab_	64.0	6.3	1.1	24.4
		H3	7.0	53.9_cd_	68.0	5.6	1.0	20.4
	E4	H1	12.8	50.0_ab_	66.3	4.6	0.5	17.8
		H2	6.1	52.2_bcd_	67.2	8.0	0.9	19.8
		H3	6.4	53.5_cd_	67.7	7.4	1.1	19.3
	E5	H1	6.1	50.6_ab_	66.3	4.2	0.7	24.6
		H2	6.3	53.4_cd_	69.6	5.0	1.3	19.8
		H3	8.9	53.8_cd_	68.1	5.1	1.0	18.9
**SEM** *L* × *E* × *H*			1.07	1.49	1.77	0.86	0.12	1.97
**P** [Table-fn t4fn4] light (*L*)			*	*	*	ns	ns	ns
**P** entry (*E*)			*	*	*	*	ns	ns
**P** harvest (*H*)			*	*	*	*	*	ns
**P** *L* × *E*			ns	ns	*	ns	ns	*
**P** *L* × *H*			ns	ns	ns	*	ns	ns
**P** *E* × *H*			*	ns	ns	ns	*	ns
**P** *L* × *E* × *H*			ns	*	ns	ns	ns	ns

1 Light condition: Low = no additional light; High = application of lamp energy.

2 *H* = harvest time point (H1 = day 116, H2 = day 133 and H3 = day 152).

3 ADF = acid detergent fiber; NDF = neutral detergent fiber; CP = crude protein; EE = ether extract; NFC = nonfiber carbohydrate.

4 ns = not significant (*P* > 0.05), * = significance (*P* < 0.05).

^a–d^Means within a column with different superscripts differ (*P* < 0.05).

As expected, the nutritional value of the stems was lower than that of the flowers and leaves, as indicated by low CP (6.6%) and high NDF (67.1%) content. The chemical composition of stems (Table 5) was affected independently by *L*, *E*, and *H*, with some observed interaction effects (*L* × *E* × *H* for the ADF content and *L* × *H* and *E* × *H* effects for the CP and EE content, respectively). Different from flowers, E1 hemp stems contained the highest CP and NFC and the lowest ADF and NDF levels compared to other hemp entries. The effect of harvest time was similar to the flowers’ nutrient content, in that the values decreased as the number of harvest times increased. Interaction effects were observed for *L* × *E* × *H* on NDF, CP, EE, and NFC values and *L* × *E* on all chemical variables of hemp stems except for the NDF content (Table 4). Similar to flowers, the only variable affected by light intensity was CP, while harvest time only showed an effect on ash content. High light intensity resulted in higher ash (8.2 vs 7.4%) and lower ADF (51.2 vs 52.5%) and NDF (66.7 vs 68.2%) concentrations of hemp stems than those that received low light intensity.

### 3.3 Cannabinoid Concentration

Quantities of cannabinoids in inflorescences for five varieties grown under the two light conditions and harvested at days 116, 133, and 152 were determined and are presented in Table 6. Light (*L*), hemp variety (*E*) and harvest time (*H*) were significant factors that impacted total CBD (36.7 vs 27.1 μg/mg high/low light; 15.1, 11.7, 58.4, 34.5, and 39.8 μg/mg for E1–E5; 33.6, 22.3, and 39.9 μg/mg for H1–H3) and total cannabinoid production (51.2 vs 39.2 μg/mg high/low light; 27.5, 18.0, 69.6, 60.9, and 50.3 μg/mg for E1–E5; 43.6, 29.5, and 62.6 μg/mg for H1–H3; the interaction of *L* × *E* and *E* × *H* was significant for these two variables as well. The photoperiod-sensitivity of the variety appears to have a strong influence on these relationships, as E1 and E2 were not influenced by changes in light intensity (26.6/28.3 and 21.1/14.8 μg/mg high/low light for total cannabinoids), unlike varieties E3, E4, and E5, which did show intensification of cannabinoid production with increased light (88.7/50.5, 67.1/54.6, and 57.3/43.4 μg/mg high/low light for total cannabinoids, demonstrating a 23–76% increase). The trifold interaction effect of *L* × *E* × *H* was significant for total cannabinoids only; the third harvest time showed the highest concentration of total cannabinoids for most varieties (62.6 μg/mg vs 43.6 and 29.5 μg/mg), particularly for those that were photoperiod-sensitive (83.8 μg/mg for H3–H5 vs 30.9 μg/mg for H1–H2, representing a 2.7-fold difference).

**Table 6:** Cannabinoid Concentration (μg/mg) of Photoperiod-Sensitive and Photoperiod-Insensitive Hemp Flowers Sampled at 116, 133, and 152 Days under Two Light Conditions

light[Table-fn t6fn1]	entry	harvest[Table-fn t6fn2]	THC-Δ^9^ total (μg/mg)[Table-fn t6fn3]	CBD total (μg/mg)[Table-fn t6fn3]	total cannabinoids (μg/mg)[Table-fn t6fn4]
low	E1	H1	0.7	27.2	34.1_bc_
		H2	0.4	2.9	6.8_a_
		H3	0.4	17.5	44_c_
	E2	H1	2.6	19.1	27.4_ab_
		H2	0.2	1.3	5.4_a_
		H3	0.3	9.7	12.7_a_
	E3	H1	1.3	67.8	83.7_e_
		H2	1.5	83.8	96_e_
		H3	1.5	72.6	86.3_e_
	E4	H1	1.0	47.8	60.5_cd_
		H2	0.7	2.8	11.3_a_
		H3	0.9	61.6	129.8_f_
	E5	H1	1.6	39.5	53.4_cd_
		H2	0.9	42.5	51.1_cd_
		H3	1.3	53.6	67.3_de_
high	E1	H1	0.7	24.7	30.7_bc_
		H2	0.6	5.2	10.7_a_
		H3	0.4	14.1	40.1_bc_
	E2	H1	2.2	17.8	24.8_ab_
		H2	2.3	1.1	10.6_a_
		H3	0.6	21.4	28_b_
	E3	H1	0.6	31.8	38.7_bc_
		H2	0.8	35.3	41.6_c_
		H3	1.3	59.3	71.2_de_
	E4	H1	2.0	35.4	46.9_c_
		H2	1.0	18.7	28.6_bc_
		H3	0.7	39.5	88.2_e_
	E5	H1	3.3	23.8	35.7_c_
		H2	0.7	28.9	34.4_c_
		H3	1.3	53.4	63.6_cd_
SEM *L* × *E* × *H*			0.53	5.85	6.02
P[Table-fn t6fn5] light (*L*)			ns	*	*
P entry (*E*)			ns	*	*
P harvest (*H*)			ns	*	*
P *L* × *E*			ns	*	*
P *L* × *H*			ns	ns	ns
P *E* × *H*			ns	*	*
P *L* × *E* × *H*			ns	ns	*

1 *H* = harvest time point (H1 = day 116, H2 = day 133, and H3 = day 152).

2 Light condition: High = application of lamp energy; Low = no additional light.

3 Total delta-9-tetrahydrocannabidiol (Δ^9^-THC) or cannabidiol (CBD) calculated as Total cannabinoid = ([acidic form] x 0.877) + [neutral form].

4 Total cannabinoids calculated as ([acidic form] x 0.877) + [neutral form] for seven cannabinoids and their acids plus THC-d8 and CBL-a.

5 ns = not significant (*P* > 0.05), * = significance (*P* < 0.05).

^a–f^Means within a column with different superscripts differ (*P* < 0.05).

## 4 Discussion

There is significant interest in using industrial hemp as a livestock feed due to its nutritional profile (optimal protein, digestible fiber, fat, and minerals);^14–16^ its availability as a local food resource, which has global sustainability implications for both large- and small-scale farmers; and provision of an economically viable option in a volatile feed procurement environment. When integrating a new material into the animal feed supply, it must be verified as safe by appropriate regulatory agencies. In the case of industrial hemp, this involves approval by the Food and Drug Administration (FDA) after an application is made containing safety data that demonstrates that hemp is safe to both the animals consuming it and that any products destined for human consumption (e.g., meat, milk, and eggs) are free of cannabinoid residues in the United States of America.

To better clarify the relationship between cannabinoid potency, light condition, and harvest time, five varieties of industrial hemp (*C. sativa*) plants were grown in four replicate blocks on a field in Imperial Valley, California, USA. This field contained artificial light sources that were turned on for 2 h every night over a period of approximately 60 days, beginning 17 days after the varieties had been seeded into the field. During the growth phase, plants were separated into two distinct treatment groups: those exposed to “high-light conditions” and those exposed to “low light conditions.” These groups were distinguished by their proximity to artificial light sources. To study the effects of harvest time, there were three sampling periods: early season sampling (day 116 after seeding), midseason sampling (day 133 after seeding), and late season sampling (day 152 after seeding). Our findings represent a single growing cycle; thus, year-to-year variability within the same location from environmental and other variables cannot be addressed or inferred from the data provided here, but we intend for this study to provide a more exhaustive examination of the agronomic, nutritive, and chemical values that impact industrial hemp’s viability as a livestock feed.

### 4.1 Agronomic and Nutritive Value

Our study’s nutritional profiling indicates that hemp offers a high nutritive value for ruminant feed, evidenced by its high CP and NFC content. The chemical composition observed is consistent with previous findings,^7,13^ showing that hemp flowers and leaves have a similar or greater nutritive value compared to common forages like high-quality alfalfa.^39^ However, the high EE (14.6%) and ash (15.1%) content in hemp flowers (which is similar to values found in other studies^19^) may limit digestibility when fed directly to ruminants, as these components may also contain cannabinoids, flavonoids, terpenes, heavy metals, and other compounds that could disrupt ruminal fermentation.^40–43^ This aligns with recent in vitro studies showing relatively low NDF and organic matter digestibility ranging from 28 ^44^ to 42%.^7^ These factors could be attenuated by limiting hemp’s percentage in the diet (e.g., setting a certain threshold for formulation in total mixed rations). However, hemp byproducts such as spent hemp biomass produced following CBD oil extraction present less of a concern due to their reduced EE content (4–7%), and have not been shown to cause adverse effects when fed to ruminants.^15,19,45,46^

In our study, the most significant effect of light intensity was on the CP content of flowers and leaves without an effect on ADF, NDF, EE, and NFC levels. Light is well-known to regulate nitrogen uptake, plant growth, and overall performance.^47–49^ Though research on light’s effect on hemp is limited, other studies have shown that higher CP content and solubility with increased light intensity, which is likely due to enhanced nitrogen uptake and photosynthetic efficiency.^47,50^ Harvest time also significantly affected the CP content of hemp flowers, with a decrease observed after the third harvest. NDF content in leaves increased after the first harvest, accompanied by a decrease in NFC. This decline in nutritive value with plant maturity is typical for forages,^51–53^ with biomass yield similarly declining after the third harvest.^54^

While hemp may not be widely grown as forage in the near future, since it is currently not competitive with other alternative forages such as kenaf (*Hibiscus cannabinus* L),^55^ hemp byproducts containing leaves and flowers could be a viable alternative to high-quality forages, potentially reducing feed costs once it is legalized. Notably, the light sensitivity trait did not consistently affect forage nutritive value, suggesting that cultivar choice and agronomic practices will depend more on the primary purpose of hemp production (e.g., fiber and CBD oil) rather than its potential as animal feed.

### 4.2 Cannabinoid Analysis Validation

Lab-to-lab analytical variation is a known phenomenon for commercial cannabinoid analysis laboratories, as is true with phytochemical assays in general. For hemp as a new commodity, the analytical methodology needs to be reproducible and generally accepted by other researchers and analytical laboratories. For this controlled production study, we determined an optimal analytical method for reporting cannabinoid concentrations across multiple production environment variables for a range of plant genetics. Both gas and liquid chromatography methods have been employed, with HPLC currently being the preferred method for analysis. There are many different HPLC methods that have been developed and published, with most being variations of an acetonitrile/water gradient on a reversed-phase C18 column. These chromatography methods are very similar and are consistently reproducible; however, variation in the results was derived from differences in sample treatment, storage, preparation, and extraction methodology. A fully standardized method for measuring cannabinoids has not yet been promulgated. Our study includes a careful examination of extraction methodology and an in-depth evaluation of our HPLC methods for method validation (Supplementary Tables 3–6).

A single-step extraction method is preferable for processing large numbers of samples and avoids losses of measurable cannabinoids that more complicated sample preparations would likely incur. Our evaluation indicates that using 100% absolute ethanol at a ratio of 20 mL to about 0.100 g of ground flower tissue provides the highest level of extractable cannabinoids. The single-step method is also preferable for processing large batches because it limits the loss of measurable cannabinoids that degrade due to more complicated sample preparation protocols and heat.

A NIST study (Exercise 2), where hemp samples were sent to various analytical laboratories, revealed that samples low in total Δ^9^-THC had greater variability than samples with high total Δ^9^-THC (Supplementary Figure 3a,b). A follow-up study (Exercise 3) that included an “oil” extract sample found a similar pattern; i.e., low Δ^9^-THC samples had significantly higher percent differences compared to high Δ^9^-THC samples (Supplementary Figure 3c,d). In both studies, percent differences for the low Δ^9^-THC samples were often greater than 50%, while total Δ^9^-THC’s relative standard deviation of reproducibility (RSDR) increased as its concentration decreased.^57,58^ These values indicate lab-to-laboratory percent differences up to 20.29% for low THC samples. The largest percentage difference for a high THC sample was 9.65%. This variability can be problematic for industrial hemp growers, as a lab could flag a sample as noncompliant if it exceeds the 0.30% total Δ^9^-THC legal threshold (U.S., dry weight) when the sample may, in fact, contain only 0.24% total THC. Thus, when establishing a method for potency compliance, it is crucial to perform interlab validation and establish standardized method parameters, ideally overseen by regulatory bodies.

### 4.3 Cannabinoid Concentrations

Overall, the cannabinoid response for varieties used in our experiment to light intensity was significant for CBD and total cannabinoids (Table 6, *L* × *E*). Numerous studies have found that cannabinoid potency is heavily dependent on the plant’s genetics, as expressed agronomically through the term genotype or variety.^56,57^ Observations are likely driven by whether the variety was photoperiod-sensitive and, thus, would respond to light intensification with increased cannabinoid production.

Harvest time on its own was found to be a significant factor affecting cannabinoid potency for CBD and total cannabinoids as well (variable *H* and *L* × *H*, Table 6). In general, hemp is a fast-growing annual plant that completes its entire growth process in 120–140 days for “full-season” or photoperiod-sensitive varieties (E1 and E2) or 70–90 days for “autoflower” or photoperiod-insensitive varieties (E3, E4, and E5).^58^ This impacts cannabinoid production as the more compressed growth window of autoflower varieties implies concentrations can change more rapidly over a given time scale as the plant reaches maturity. A recent study by Burgel et al. investigated how different growth stages affected cannabinoid potency in seven varieties of industrial hemp. They discovered an overall trend of declining CBD potency over increasingly older growth stages, although they noted a substantial amount of variability in CBD concentration and response to growth stage across varieties·^57^ This variability by variety for CBD was seen in the current work (Table 6, 1.1–83.8 μg/mg CBD and 5.4–129.8 μg/mg total cannabinoids), with harvest time generally showing the highest concentration of cannabinoids during the third and final harvest. When the interaction of all three variables was conserved (*L* × *E* × *H*), only the total cannabinoid concentration remained significant. This measurement also took into consideration the other five cannabinoids (neutral and acidic forms of CBG, CBDV, THCV, CBN, and CBC plus CBLA and Δ^8^-THC) that were evaluated, which could have influenced the statistical outcome.

To further explore this idea, consider the study by Ingallina et al. on cultivation time and cannabinoid potency in industrial hemp that was conducted in Italy. Researchers investigated the potency of six cannabinoids in four varieties of commercially available industrial hemp once a month over a 4-month period (June to September).^59^ They found a general trend in which Δ^9^-THC and CBD concentrations increased over increasingly later harvest periods. Although this does contrast with some of the data found here, it introduces the importance of considering the scale. Ingallina et al. had harvest periods over a much longer time range, with their first and last harvest periods being 4 months apart. In this study, the first and last harvest periods are only approximately 1 month apart (36 days); thus, it is likely that the difference in time scale is responsible for some of the discrepancies between the two studies. Season of harvest should also be considered, as the concept of changing and related abiotic factors influences plant secondary metabolite production. This demonstrates that both the diversity and amount of cannabinoids expressed in *C. sativa* are influenced by environmental variables as well as genotypes, which have interactive effects on the ultimate chemoprofile that is produced.^60–63^

In conclusion, the light sensitivity of the hemp varieties, light intensity applied, and harvest time significantly influenced agronomic and nutritional characteristics as well as the cannabinoid profile of hemp grown under the conditions described in this study. High light intensity increased agronomic and nutritional variables, particularly CP and NFC, but the magnitude of the response varied by hemp genotype, dependent on whether the variety was photoperiod-sensitive or not. Cannabinoid concentrations increased in the three photosensitive hemp varieties when grown under high light intensity, while the latter harvest time demonstrated the highest values in all varieties overall. Thus, photosensitive varieties come with the trade-off of being more desirable from a nutritive standpoint but riskier in potential for cannabinoid accumulation.

Being aware of both natural environmental and externally introduced cultivation factors can help guide scientists in creating experimental designs that will effectively address their research questions to determine conditions for optimal hemp production with the intent of being integrated into the animal or human food chain. Being familiar with each of these aspects will also be beneficial to hemp growers and producers, as it will allow them to have access to and confidently select compliant hemp varieties while cultivating efficient agronomic practices that will most influence their specific crop goals. Current indications from this and other studies are that industrial hemp is a viable, sustainable feed alternative if risks around cannabinoid production can be managed through timing of harvest and variety selection, which should be considered for approval by regulatory bodies, particularly in light of the emergent food security needs of our global human population. To achieve this aim, additional research is needed to continue to tease apart the complex and diverse relationships between *C. sativa* plants and the elements that influence growth, cannabinoid expression, and other important metrics of hemp cultivation. Further, continuing to assimilate information through in vivo feeding trials in ruminant livestock species is warranted to evaluate implications on digestion and general production parameters. Altogether, this work should culminate in the identification of forage varieties of hemp, similar to recommendations of forage grasses for livestock managers, which are developed based on factors such as yield, persistence, digestibility, and adaptability to specific climates.^64^

## Supplementary Material



## Notes

Figures—Field map layout, Extraction method comparison, NIST CannaQAP Exercise results, Agronomic variables significant for three-way interaction, Nutritive variables significant for three-way interaction; Tables—Local weather data, Hemp variety description, Solvent and extraction volume comparison, Soxhlett vs sonication extraction, Extraction solvent comparison, Method validation parameters (PDF)

## #

Universitas Sebelas Maret, Surakarta, Central Java 57216, Indonesia

## ∇

Mori, Cambridge Crops, Inc., d/b/a, 500 Rutherford Avenue Ste. 105, Boston, MA 02129, United States.

## Abbreviations

ADF: acid detergent fiber
ANOVA: analysis of variance
CBC: cannabichromene
CBCA: cannabichromene acid
CBCVA: cannabichromevarinic acid
CBD: cannabidiol
CBDA: cannabidiolic acid
CBDV: cannabivarin
CBDVA: cannabivarinic acid
CBG: cannabigerol
CBGA: cannabigerolic acid
CBGVA: cannabigerovarinic acid
CBL: cannabicyclol
CBLA: cannabicyclolic acid
CBN: cannabinol
CBNA: cannabinolic acid
CBV: cannabivarin
DMSO: dimethylsulfoxide
EE: ether extract
FDA: food and drug administration
HPLC: high-performance liquid chromatography
LOD: limits of detection
LOQ: limit of quantitation
NDF: neutral detergent fiber
NFC: nonfiber carbohydrate
RSD: relative standard deviation
RSDR: relative standard deviation of reproducibility
Δ^8^-THC: delta-8-tetrahydrocannibinol
Δ^9^-THC: delta-9-tetrahydrocannibinol
THCA: delta-9-tetrahydrocannibinolic acid
THCV: tetrahydrocannabivarin
THCVA: tetrahydrocannabivarinic acid
UPLC-DAD: ultra high performance-diode array detection
USA: United States of America

## References

[1] United States of America Congress. H.R.5485—115th Congress (2017–2018): Hemp Farming Act of 2018, 2026 https://www.congress.gov/bill/115th-congress/house-bill/5485 (accessed 2026-03-05).

[2] Viskovic J., Zheljazkov V. D., Sikora V., Noller J., Latkovic D., Ocamb C. M., Koren A. (2023). Industrial Hemp (*Cannabis
Sativa* L.) Agronomy and Utilization: A Review. Agron.-Basel.

[3] Gulck T., M?ller B. L. (2020). Phytocannabinoids: Origins and Biosynthesis. Trends Plant Sci..

[4] Tanney C. A. S., Backer R., Geitmann A., Smith D. L. (2021). Cannabis
Glandular
Trichomes: A Cellular Metabolite Factory. Front.
Plant Sci..

[5] Jin D., Dai K., Xie Z., Chen J. (2020). Secondary Metabolites Profiled in
Cannabis inflorescences, Leaves, Stem Barks, and Roots for Medicinal
Purposes. Sci. Rep..

[6] Ross S. A., Mehmedic Z., Murphy T. P., Elsohly M. A. (2000). GC-MS Analysis of
the Total Delta9-THC Content of Both Drug- and Fiber-Type Cannabis
Seeds. J. Anal. Toxicol..

[7] Kleinhenz M. D., Magnin G., Ensley S. M., Griffin J. J., Goeser J., Lynch E., Coetzee J. F. (2020). Nutrient
Concentrations, Digestibility,
and Cannabinoid Concentrations of Industrial Hemp Plant Components. Appl. Anim. Sci..

[8] Mohamed N., Slaski J. J., Shwaluk C., House J. D. (2024). Chemical Characterization
of Hemp (*Cannabis Sativa* L.)-Derived Products and
Potential for Animal Feed. *ACS FOOD*. Sci. Technol..

[9] Irawan A., Puerto-Hernandez G. M., Ford H. R., Busato S., Ates S., Cruickshank J., Ranches J., Estill C. T., Trevisi E., Bionaz M. (2024). Feeding Spent
Hemp Biomass to Lactating Dairy Cows:
Effects on Performance, Milk Components and Quality, Blood Parameters,
and Nitrogen Metabolism. J. Dairy Sci..

[10] Irawan A., Nosal D. G., Muchiri R. N., van Breemen R. B., Ates S., Cruickshank J., Ranches J., Estill C. T., Thibodeau A., Bionaz M. (2025). Cannabinoid Distribution and Clearance
in Feeding Spent Hemp Biomass to Dairy Cows and the Potential Exposure
to Δ9-THC by Consuming Milk. J. Agric.
Food Chem..

[11] Irawan A., Muchiri R. N., Parker N. B., van Breemen R. B., Ates S., Bionaz M. (2024). Cannabinoid Residuals in Tissues
of Lambs Fed Spent Hemp Biomass and Consumer’s Exposure Assessment. FOOD Chem. Toxicol..

[12] Smith D. J., Serum E. M., Winders T. M., Neville B., Herges G. R., Dahlen C. R., Swanson K. C. (2023). Excretion and Residue
Depletion of
Cannabinoids in Beef Cattle Fed Hempseed Cake for 111 Days. Food Addit. Contam. Part A.

[13] Wagner B., Gerletti P., Fürst P., Keuth O., Bernsmann T., Martin A., Schäfer B., Numata J., Lorenzen M. C., Pieper R. (2022). Transfer of Cannabinoids
into the Milk of Dairy Cows
Fed with Industrial Hemp Could Lead to Δ9-THC Exposure That
Exceeds Acute Reference Dose. Nat. Food.

[14] Altman A., Klotz J., Mcleod K., Vanzant E., Harmon D. (2024). Review: Utilizing
Industrial Hemp (*Cannabis Sativa* L.) by-Products
in Livestock Rations. Anim. FEED Sci. Technol..

[15] Parker N. B., Bionaz M., Ford H. R., Irawan A., Trevisi E., Ates S. (2022). Assessment of Spent
Hemp Biomass as a Potential Ingredient in Ruminant
Diet: Nutritional Quality and Effect on Performance, Meat and Carcass
Quality, and Hematological Parameters in Finishing Lambs. J. Anim. Sci..

[16] Xu Y., Li J., Zhao J., Wang W., Griffin J., Li Y., Bean S., Tilley M., Wang D. (2021). Hempseed as a Nutritious
and Healthy Human Food or Animal Feed Source: A Review. Int. J. FOOD Sci. Technol..

[17] Drewery M., Hustvedt G. (2024). Consumer Support for
Hemp By-Products as Food and Feed. J. Nat. FIBERS.

[18] Ates, A. M. ; Liefert, O. Feed Outlook: USDA, 2024 http://www.ers.usda.gov/publications/pub-details/?pubid=108285 (accessed 2024-05-21).

[19] Irawan A., Buffington H., Ates S., Bionaz M. (2025). Use of Industrial Hemp
Byproducts in Ruminants: A Review of the Nutritional Profile, Animal
Response, Constraints, and Global Regulatory Environment. J. Cannabis Res..

[20] Abernethy, A. Hemp Production and the 2018 Farm Bill. Food and Drug Administration, 2021. https://www.fda.gov/news-events/congressional-testimony/hemp-production-and-2018-farm-bill-07252019 (accessed 2021-11-12).

[21] American Veterinary Medical Association. Organizations warn against hemp in pet food, livestock feed. American Veterinary Medical Association, 2023 https://www.avma.org/news/organizations-warn-against-hemp-pet-food-livestock-feed (accessed 2023-09-11).

[22] USDA. Hemp Production | Agricultural Marketing Service. United States Department of Agriculture (USDA), 2021. https://www.ams.usda.gov/rules-regulations/hemp (accessed 2021-11-12).

[23] EFSA
Panel on Additives and Products or Substances used in Animal Feed
(FEEDAP) (2011). Scientific Opinion
on the Saftey of Hemp (Cannabis Genus) for Use as Animal Feed. EFSA J..

[24] USGS. Imperial Valley, California, USA | Earth Resources Observation and Science (EROS) Center. United States Geologicals Survey (USGS), 2021. https://eros.usgs.gov/image-gallery/earthshot/imperial-valley-california-usa (accessed 2021-11-12).

[25] NRCS. NRCS Soils, Soil Surveys by State | Imperial County, Imperial Valley Area, 2021 https://www.nrcs.usda.gov/wps/portal/nrcs/surveylist/soils/survey/state/?stateId=CA (accessed 2021-11-12).

[26] WRCC. Imperial, California—Climate Summary. Western Regional Climate Center (WRCC), 2021. https://wrcc.dri.edu/cgi-bin/cliMAIN.pl?caimpe+sca (accessed 2021-11-12).

[27] AOAC International . Official Methods of Analysis of AOAC International, 20th ed.; AOAC International: Gaithersburg, MD, 2016.

[28] Hall, M. B. ; Calculation of Non-Neutral Detergent Fiber Carbohydrate Content of Feeds That Contain Non-Protein Nitrogen. Report No.: EDIS No. 339; Institute of Food and Agriculture Sciences, University of Florida Cooperative Extension Service: Gainesville, FL, USA, 2001.

[29] Van
Soest P. J., Robertson J. B., Lewis B. A. (1991). Methods for Dietary
Fiber, Neutral Detergent Fiber, and Nonstarch Polysaccharides in Relation
to Animal Nutrition. J. Dairy Sci..

[30] Lewis M. M., Yang Y., Wasilewski E., Clarke H. A., Kotra L. P. (2017). Chemical
Profiling of Medical Cannabis Extracts. ACS
Omega.

[31] Christinat N., Savoy M.-C., Mottier P. (2020). Development, Validation
and Application
of a LC-MS/MS Method for Quantification of 15 Cannabinoids in Food. Food Chem..

[32] Meregalli M. M., Puton B. M. S., Camera F. D., Amaral A. U., Zeni J., Cansian R. L., Mignoni M. L., Backes G. T. (2020). Conventional and
Ultrasound-Assisted Methods for Extraction of Bioactive Compounds
from Red Araçá Peel (Psidium Cattleianum Sabine). Arab. J. Chem..

[33] Samavardhana K., Supawititpattana P., Jittrepotch N., Rojsuntornkitti K. (2015). Effects of
Extracting Conditions on Phenolic Compounds and Antioxidant Activity
from Different Grape Processing Byproducts. Int. Food Res. J..

[34] Thompson M., Ellison S. L. R., Wood R. (2002). Harmonized Guidelines for Single-Laboratory
Validation of Methods of Analysis (IUPAC Technical Report). Pure Appl. Chem..

[35] Masson-Matthee, M. D. The Codex Alimentarius Commission and Its Standards; T.M.C. Asser Press; Distributed by Cambridge University Press: The Hague: West Nyack, NY, 2007.

[36] Lee M. J., Lee J. M., Kim S., Kim H. J. (2019). Simultaneous Analysis
and Measurement of Uncertainty Estimation of Six Isoflavones in Cheonggukjang
by Liquid Chromatography-Electrospray Tandem Mass Spectrometry. Food Chem..

[37] US FDA . Field Science—ORA Laboratory Manual; FDA, 2023.

[38] Berthold E. C., Yang R., Sharma A., Kamble S. H., Kanumuri S. R., King T. I., Popa R., Freeman J. H., Brym Z. T., Avery B. A., McCurdy C. R. (2020). Regulatory
Sampling of Industrial
Hemp Plant Samples (*Cannabis* Sativa L.) Using UPLC-MS/MS
Method for Detection and Quantification of Twelve Cannabinoids. J. Cannabis Res..

[39] Lynch J. P., Jin L., Church J. S., Baah J., Beauchemin K. A. (2015). Fibrolytic
Enzymes and a Ferulic Acid Esterase-Producing Bacterial Additive Applied
to Alfalfa Hay at Baling: Effects on Fibre Digestibility, Chemical
Composition and Conservation Characteristics. Grass Forage Sci..

[40] Benelli G., Pavela R., Petrelli R., Cappellacci L., Santini G., Fiorini D., Sut S., Dall’Acqua S., Canale A., Maggi F. (2018). The Essential Oil from Industrial
Hemp (*Cannabis* Sativa L.) by-Products as an Effective
Tool for Insect Pest Management in Organic Crops. Ind. Crops Prod..

[41] Fiorini D., Scortichini S., Bonacucina G., Greco N., Mazzara E., Petrelli R., Torresi J., Maggi F., Cespi M. (2020). Cannabidiol-Enriched
Hemp Essential Oil Obtained by an Optimized Microwave-Assisted Extraction
Using a Central Composite Design. Ind. Crops
Prod..

[42] Flores-Sanchez I. J., Verpoorte R. (2008). Secondary
Metabolism in Cannabis. Phytochem. Rev..

[43] Valizadehderakhshan M., Shahbazi A., Kazem-Rostami M., Todd M. S., Bhowmik A., Wang L. (2021). Extraction of Cannabinoids
from *Cannabis* Sativa
L. (Hemp)—Review. Agriculture.

[44] Vastolo A., Calabro S., Pacifico S., Koura B. I., Cutrignelli M. I. (2021). Chemical
and Nutritional Characteristics of *Cannabis* Sativa
L. Co-Products. J. Anim. Physiol. Anim. Nutr..

[45] Irawan A., Puerto-Hernandez G. M., Ford H. R., Busato S., Ates S., Cruickshank J., Ranches J., Estill C. T., Trevisi E., Bionaz M. (2023). Feeding Spent
Hemp Biomass to Lactating Dairy Cows:
Effects on Performance, Milk Components and Quality, Blood Parameters,
and Nitrogen Metabolism. J. Dairy Sci..

[46] Meador M. A., Ates S., Kutzler M. A. (2024). Feeding Spent Hemp Biomass Does Not
Adversely Affect Fertility in Rams. Am. J. Vet.
Res..

[47] Esmaeili S., Aliniaeifard S., Dianati Daylami S., Karimi S., Shomali A., Didaran F., Telesiński A., Sierka E., Kalaji H. M. (2022). Elevated
Light Intensity Compensates for Nitrogen Deficiency during Chrysanthemum
Growth by Improving Water and Nitrogen Use Efficiency. Sci. Rep..

[48] Poorter H., Niinemets Ü., Ntagkas N., Siebenkäs A., Mäenpää M., Matsubara S., Pons T. (2019). A Meta-Analysis of Plant Responses to Light Intensity for 70 Traits
Ranging from Molecules to Whole Plant Performance. New Phytol..

[49] Xu J., Guo Z., Jiang X., Ahammed G. J., Zhou Y. (2021). Light Regulation of
Horticultural Crop Nutrient Uptake and Utilization. Hortic. Plant J..

[50] Song J., Huang H., Hao Y., Song S., Zhang Y., Su W., Liu H. (2020). Nutritional
Quality, Mineral and Antioxidant Content
in Lettuce Affected by Interaction of Light Intensity and Nutrient
Solution Concentration. Sci. Rep..

[51] Bekewe P. E., Castillo M. S., Acosta J. J., Rivera R. (2020). Defoliation Management
Effects on Nutritive Value of ‘Performer’ Switchgrass. Crop Sci..

[52] Coblentz W. K., Ottman M. J. (2022). Effects of Harvest
Date and Growth Stage on Triticale
Forages in the Southwest USA: Kinetics of in Vitro Disappearance of
Fiber and Dry Matter. J. Anim. Sci..

[53] Baber K. (2020). The Effects
of Defoliation on the Nutritive Value of Common Forage Grasses. Nat. Sci. Educ..

[54] Kolodziej J., Pudelko K., Mankowski J. (2023). Energy and Biomass Yield of Industrial
Hemp (*Cannabis* Sativa L.) as Influenced by Seeding
Rate and Harvest Time in Polish Agro-Climatic Conditions. J. Nat. Fibers.

[55] Stringer, C. Evaluating Hemp (Cananbis Sativa) as a Forage Based on Yield, Nutritive Analysis, and Morphological Composition, University of Kentucky, Theses and Dissertations, 2018. 10.13023/etd.2018.235.

[56] van
Velzen R., Schranz M. E. (2021). Origin and Evolution of the Cannabinoid
Oxidocyclase Gene Family. Genome Biol. Evol..

[57] Burgel L., Hartung J., Pflugfelder A., Graeff-Hönninger S. (2020). Impact of
Growth Stage and Biomass Fractions on Cannabinoid Content and Yield
of Different Hemp (*Cannabis* Sativa L.) Genotypes. Agronomy.

[58] Roseberg, R. Optimum irrigation for CBD-type hemp plants in the field. Oregon State University Extension Service, 2026 https://extension.oregonstate.edu/catalog/em-9571-optimum-irrigation-cbd-type-hemp-plants-field (accessed 2026-03-18).

[59] Ingallina C., Sobolev A. P., Circi S., Spano M., Fraschetti C., Filippi A., Di Sotto A., Di Giacomo S., Mazzoccanti G., Gasparrini F., Quaglio D., Campiglia E., Carradori S., Locatelli M., Vinci G., Rapa M., Ciano S., Giusti A. M., Botta B., Ghirga F., Capitani D., Mannina L. (2020). *Cannabis* Sativa
L. inflorescences from Monoecious Cultivars Grown in Central Italy:
An Untargeted Chemical Characterization from Early Flowering to Ripening. Molecules.

[60] Abdollahi M., Sefidkon F., Peirovi A., Calagari M., Mousavi A. (2021). Assessment
of the Cannabinoid Content from Different Varieties of *Cannabis* Sativa L. during the Growth Stages in Three Regions. Chem. Biodivers..

[61] De
Prato L., Ansari O., Hardy G. E. S. J., Howieson J., O’Hara G., Ruthrof K. X. (2022). The Cannabinoid
Profile and Growth of Hemp (*Cannabis* Sativa L.) Is
Influenced by Tropical Daylengths and Temperatures, Genotype and Nitrogen
Nutrition. Ind. CROPS Prod..

[62] Linder E. R., Young S., Li X., Henriquez Inoa S., Suchoff D. H. (2022). The Effect of Harvest Date on Temporal
Cannabinoid
and Biomass Production in the Floral Hemp (*Cannabis* Sativa L.) Cultivars BaOx and Cherry Wine. Horticulturae.

[63] Noppawan P., Bainier C., Lanot A., McQueen-Mason S., Supanchaiyamat N., Attard T. M., Hunt A. J. (2022). Effect of Harvest
Time on the Compositional Changes in Essential Oils, Cannabinoids,
and Waxes of Hemp (*Cannabis* Sativa L.). R. Soc. Open Sci..

[64] Shewmaker, G. ; Bohle, M. ; Pasture and Grazing Management in the Northwest; PNW 614, 2023.

