# Supplementary material for: Nutritive Value and Cannabinoid Potency of Diverse Hemp (Cannabis sativa L.) Varieties Grown under Different Light Conditions and Harvested across Multiple Time Points as a Possible Feed Source for Livestock
Source: J Agric Food Chem. 2026 Apr 30;74(18):14297–310. doi: 10.1021/acs.jafc.5c16989 (PMC13178076; doi:10.1021/acs.jafc.5c16989)
Supplement: Supplementary file 1 [file jf5c16989_si_001.pdf]

Supporting information

Nutritive value and cannabinoid potency of diverse hemp (*Cannabis sativa* L.) varieties grown under different light conditions and harvested across multiple time points as a possible feed source for livestock

Jennifer M. Duringer,[1] Ashley Saindon,[1] Agung Irawan,[2¥] Serkan Ates,[2] Korey J. Brownstein,[3†] Kelly M. Gude,[3†§] Chris Ringo,[4] Chad Alan Kinney,[5] Mark Berhow,[3†] Jeffrey Steiner[4]*

[1]Department of Environmental & Molecular Toxicology, 139 Oak Creek Building, Oregon State University, Corvallis, OR 97333, USA

[2]Department of Animal and Rangeland Sciences, Oregon State University, Corvallis, OR 97331, USA

[3]USDA, ARS, NCAUR, Functional Foods Research Unit, Peoria, IL 61604, USA

[4]Global Hemp Innovation Center, Oregon State University, Corvallis, OR 97331, USA

[5]Institute for Cannabinoid Research, Colorado State University, Pueblo, CO 81001, USA

[*]Corresponding author, Email: Jennifer.Duringer@oregonstate.edu

[¥]Current mailing address: Universitas Sebelas Maret, Surakarta, Central Java 57216, Indonesia

[§]Current mailing address: Mori, Cambridge Crops, Inc., d/b/a, 500 Rutherford Avenue Ste.105, Boston, MA 02129, USA

Figure S1. Field layout showing randomized complete block design of the experiment. Red diamonds indicate placement of light fixtures.

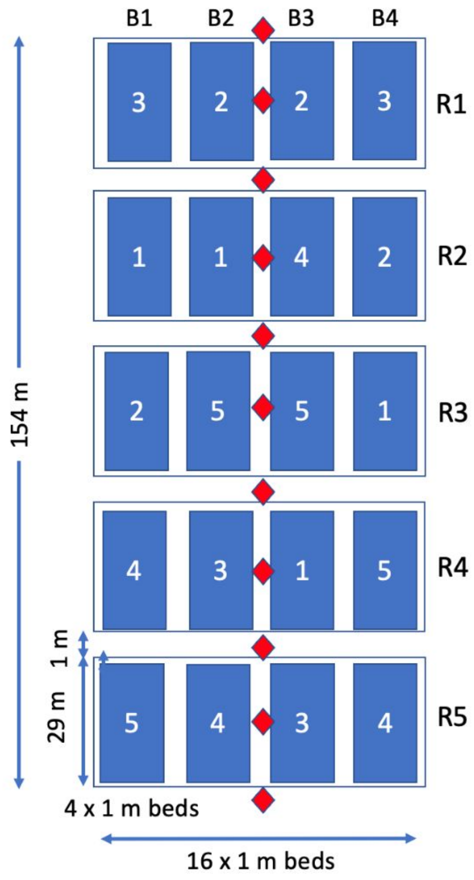

Figure S2. Comparison of the automated 20-cycle Soxhlet extractor method to the single-step sonication method. The percent totals for Δ^9-THC (a), cannabinoids (b), CBD (c), and CBG (d), as well as the individual percentages of CBDA (e) and CBD (f) in the extracts, as measured by UPLC-DAD at 228 nm.

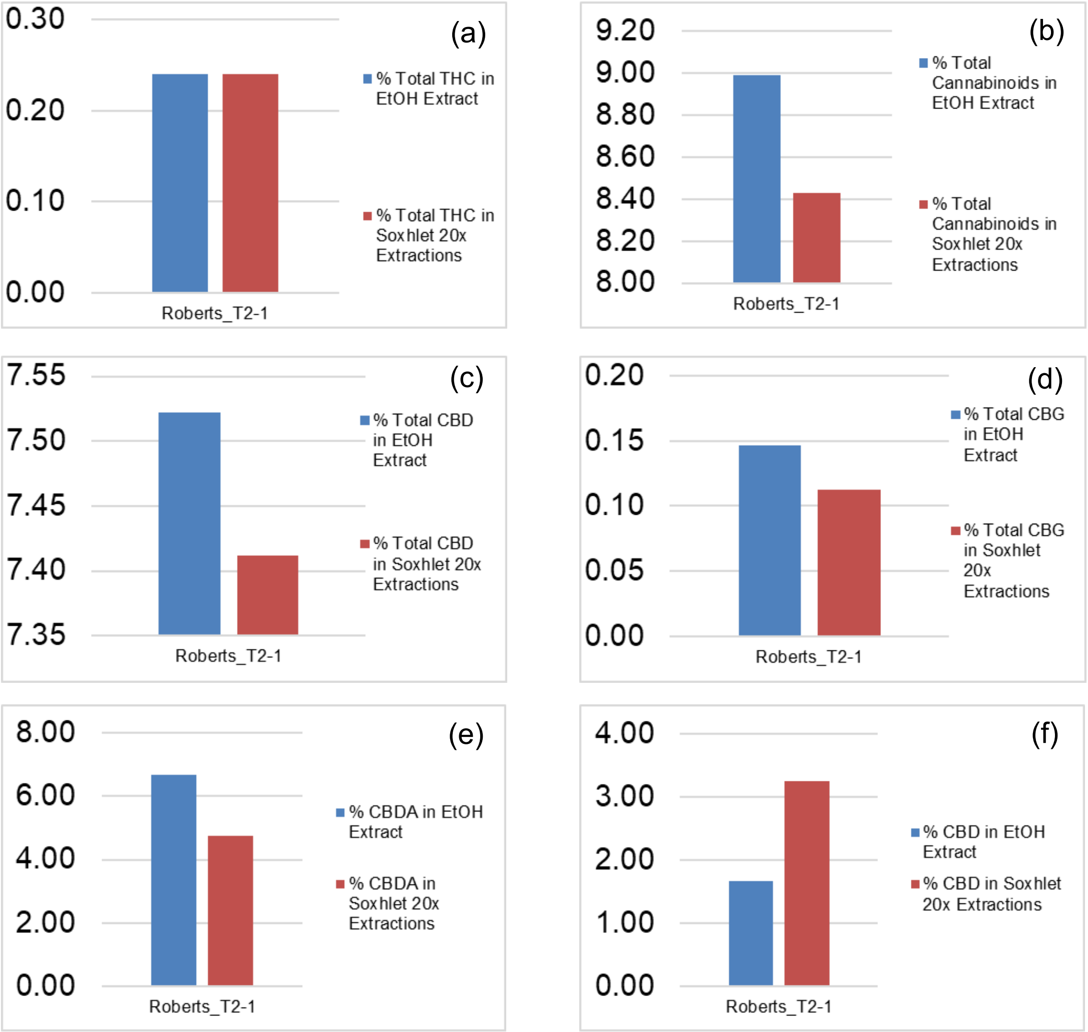

Figure S3. Analysis of percent total Δ^9-THC data from NIST CannaQAP Exercise 2 (a, b) and Exercise 3 (c, d) samples (Wilson and Abdur-Rahman 2022). Both low (a, c) and high (b, d) Δ^9-THC samples were investigated. The × and horizontal line in each box plot indicate the mean and median, respectively. In (a) and (c), each point represents an individual outlier. OS corresponds to "oil" extract sample, while PS corresponds to plant sample.

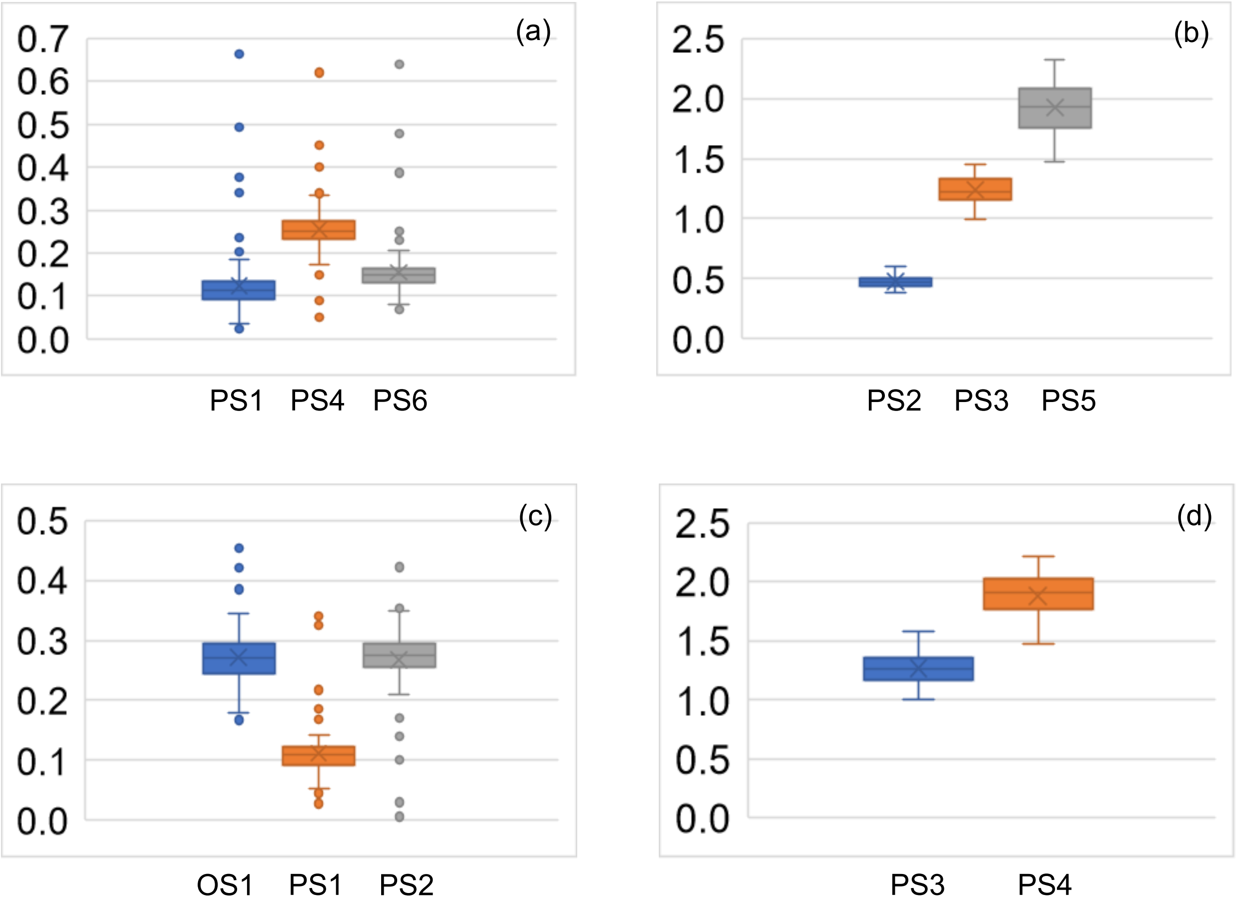

Figure S4. Agronomic characteristics of photoperiod-sensitive and photoperiod-insensitive hemp flowers sampled at 116, 133 and 152 days under two light conditions significant for **E**ntry x **L**ight x **H**arvest interaction. Compact letter display values above bars, bars sharing a letter are not significant different (α = 0.05); L=light (L1=no additional light, L2=application of lamp energy); H=harvest time point (H1=day 116, H2=day 133 and H3 = day 152).

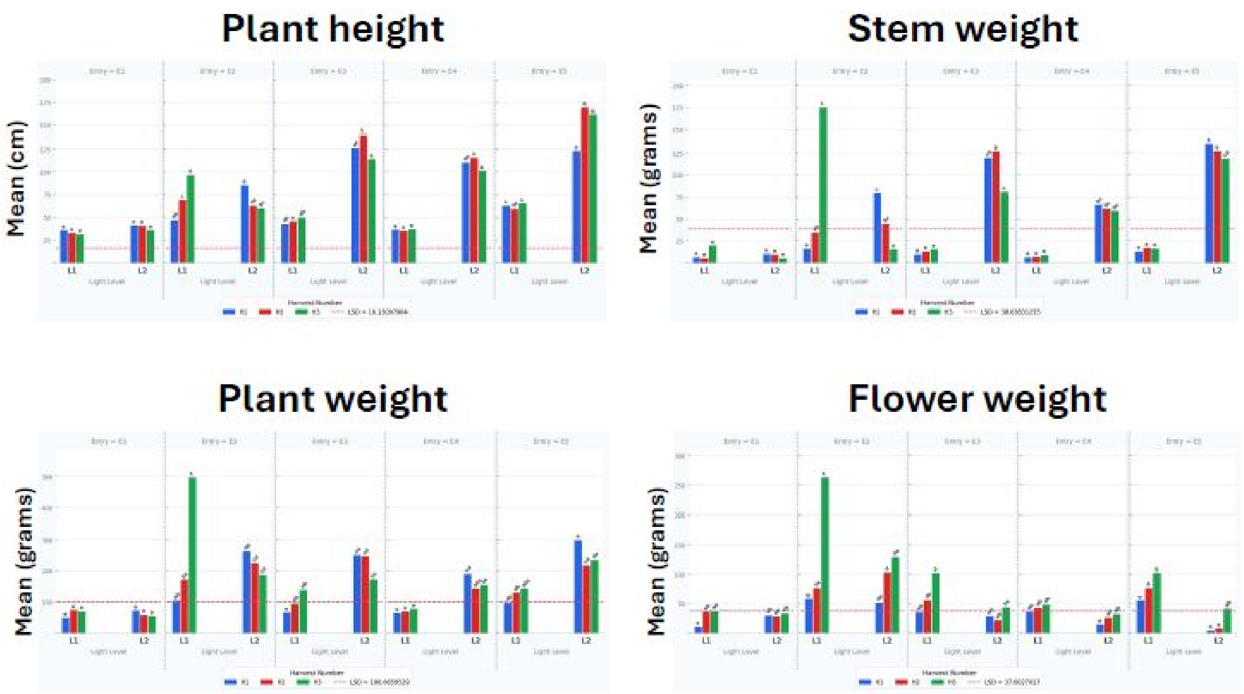

Figure S5. Nutritive values of photoperiod-sensitive and photoperiod-insensitive hemp flowers, leaves and stems sampled at 116, 133 and 152 days under two light conditions significant for **E**ntry x **L**ight x **H**arvest interaction. Compact letter display values above bars, bars sharing a letter are not significant different (α = 0.05); L=light (L1=no additional light, L2=application of lamp energy); H=harvest time point (H1=day 116, H2=day 133 and H3 = day 152).

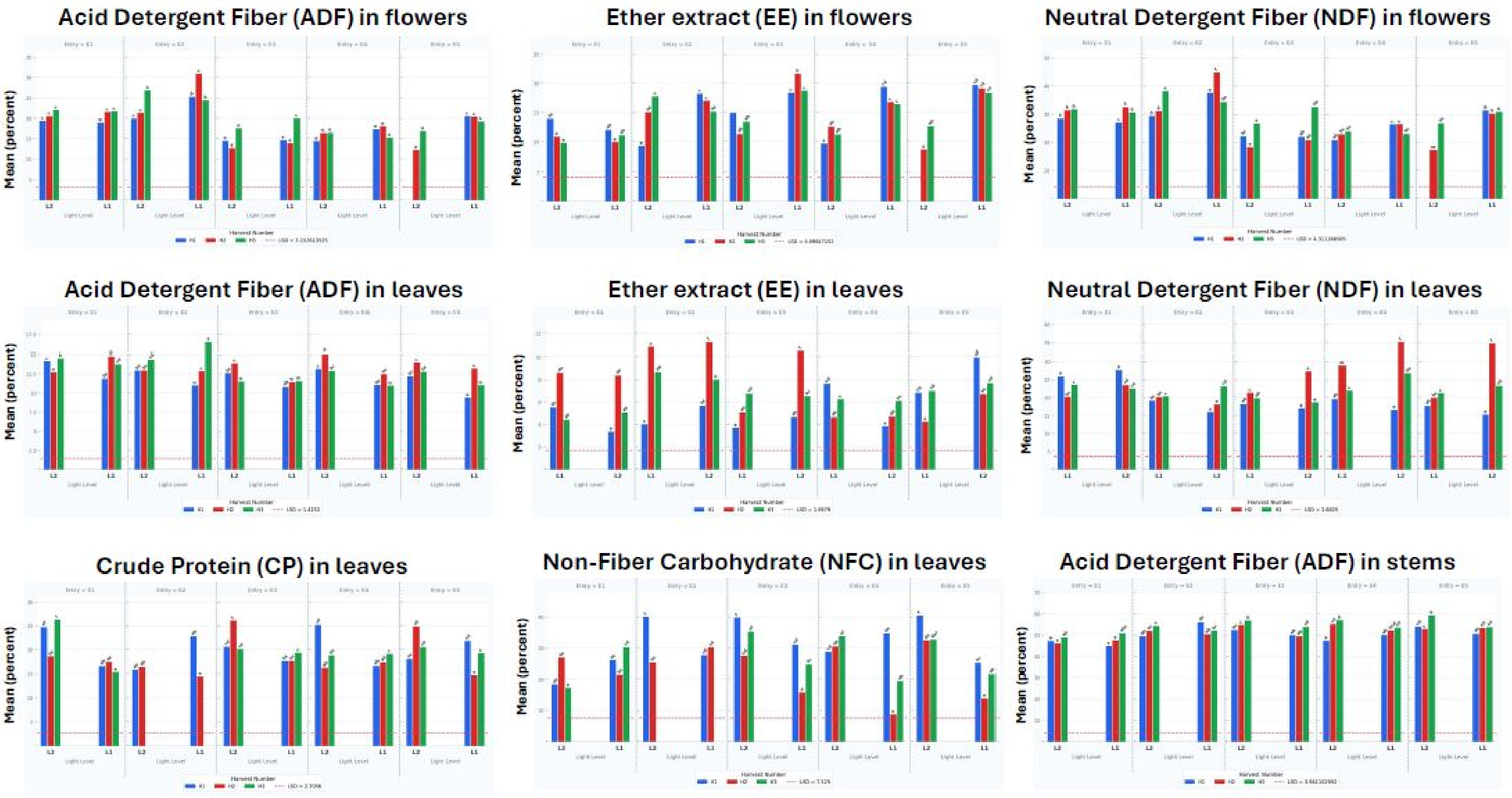

Table S1. Comparison of extraction solvents and volume.

Fraction[a]	Solvent[b]	Total Cannabinoids (%)	Total CBD (%)	Total Δ^9-THC (%)
**Extract 1+2**	**1. 200x EtOH (sonicate)**	**16.13**	**12.65**	**0.45**
Extract 1+2	2. 200x ACN (sonicate)	13.81	10.94	0.37
Extract 1+2	3. 200x MeOH (sonicate)	14.29	11.15	0.39
Extract 1+2	4. 200x 80:20 MeOH:H2O (sonicate)	12.79	10.45	0.35
Extract 1+2	5. 200x DMSO/MeOH (sonicate)	15.01	11.77	0.41
Extract 1+2	6. 200x EtOH (orbital shaker)	13.53	10.60	0.34
Extract 1+2	7. 200x ACN + 0.1% FA (sonicate)	14.13	11.08	0.39
Extract 3	1. 200x EtOH (sonicate)	0.02	0.02	0.00
Extract 3	2. 200x ACN (sonicate)	0.02	0.02	0.00
Extract 3	3. 200x MeOH (sonicate)	0.03	0.03	0.00
Extract 3	4. 200x 80:20 MeOH:H2O (sonicate)	0.06	0.06	0.00
Extract 3	5. 200x DMSO/MeOH (sonicate)	0.03	0.03	0.00
Extract 3	6. 200x EtOH (orbital shaker)	0.03	0.03	0.00
Extract 3	7. 200x ACN + 0.1% FA (sonicate)	0.03	0.03	0.00

[a]Samples were extracted in sequence three times. Extracts 1 and 2 were combined; the third extraction was run separately. All extractions used the same sample.

[b]EtOH=ethanol, ACN=acetonitrile, MeOH=methanol, H2O=water, DMSO=dimethylsulfoxide, FA=formic acid

Table S2. Comparison of extraction solvents using multiple cultivars (N=3, performed in triplicate).

Solvent	Total Cannabinoids (%)	CBD-A (%)	THC-A (%)	Total CBD (%)	Total Δ^9-THC (%)
Methanol	7.64	7.04	0.15	7.22	0.19
**Ethanol**	**7.99**	**7.30**	**0.17**	**7.50**	**0.21**
Acetonitrile	7.10	6.32	0.17	6.66	0.21

Table S3. Comparison of Soxhlett extraction for 20 cycles (1 gram by 200 mL ethanol total volume) to single step extraction (20 mL ethanol for 100 mg dried sample).

Extraction Method	Total Cannabinoids (%)	Total CBD (%)	Total CBG (%)	Total Δ^9-THC (%)
**Sonication 18 h**	**11.53**	**10.51**	**0.16**	**0.27**
Soxhlett 20x	11.57	10.32	0.12	0.33

Table S4. Method validation parameters.

Cannabinoid	RT[a]	Slope	Intercept	R^2	LOD (ug/mg)[b]	LOQ (ug/mg)[c]	RSD (%)[d]	$U = k$ * RSD[e]
**CBDV-A**	2.119	82.374	0.571	0.9956	0.002	0.005	5.196	11.140
**CBDV**	2.449	90.430	0.000	0.9928	0.001	0.004	5.662	12.139
**CBD-A**	3.078	103.86	0.072	0.9999	0.001	0.004	7.897	16.932
**CBG-A**	3.269	76.666	0.526	0.9884	0.002	0.005	5.662	12.139
**CBG**	3.416	84.251	0.000	0.994	0.001	0.005	5.267	11.293
**CBD**	3.505	80.708	0.000	0.9934	0.002	0.005	5.378	11.531
**THCV**	3.616	78.489	0.000	0.9936	0.002	0.005	5.346	11.462
**THCV-A**	3.731	73.809	0.472	0.9931	0.002	0.005	5.543	11.885
**CBN-A***	4.339	62.064	0.336	0.9938	0.002	0.008	5.875	12.595
**CBN***	4.350	197.15	0.000	0.9934	0.001	0.003	5.293	11.349
**Δ^9-THC**	5.044	75.083	0.000	0.9933	0.002	0.006	5.479	11.748
**Δ^8-THC**	5.223	65.863	0.000	0.9933	0.002	0.008	5.754	12.336
**THC-A**	5.574	70.281	0.489	0.9889	0.002	0.007	5.274	11.307
**CBL**	5.728	52.836	0.000	0.9916	0.002	0.010	5.522	11.840
**CBC**	5.945	167.32	0.000	0.9933	0.001	0.003	5.395	11.567
**CBC-A**	6.083	28.852	0.175	0.9906	0.004	0.023	7.524	16.132
**CBL-A**	6.246	45.888	0.677	0.9969	0.003	0.011	5.212	11.175

[a]RT = retention time

[b]LOD (Limit of Detection) = 3.3 x SD /Slope (SD: standard deviation) (Lee et al., 2019)

[c]LOQ (Limit of Quantification) = 10 x SD /Slope (SD: standard deviation) (Lee et al., 2019).

[d]*RSD (%)* = Percent relative standard deviation, intra-day precision.

[e]$U = k$ * RSD provided by the FDA Office of Regulatory Affairs (ORA Laboratory Manual, 2019). The relative standard deviation used in this calculation was the one generated from the intra-day validation. With a 95% confidence level, a coverage factor of k =2.144 for $N = 15$ was used (Berthold et al., 2020).

Table S5. Weather data for Imperial, California (USA) during months experiment was conducted[a]

	Dec 2019	Jan 2020	Feb 2020	Mar 2020	Apr 2020
Avg. temp	54.80	57.90	60.40	62.20	72.10
Min. temp	44.70	44.80	46.60	51.20	57.90
Max temp.	64.80	71.00	74.00	73.20	86.20
Precipitation (in.)	0.59	0.02	0.46	1.78	0.38
1901-2000 avg. temp	54.47	54.17	58.44	63.09	69.84
Historic low temp	35.00	32.50	38.30	42.30	49.60
Historic high temp	75.10	76.90	80.40	88.70	92.90
1901-2000 avg. precipitation (in)	0.55	0.53	0.50	0.37	0.10

[a]Information obtained from the Western Regional Climate Center, https://wrcc.dri.edu/cgi-bin/cliMAIN.pl?caimpe+sca

Table S6. Description of hemp varieties used in evaluation of agronomic, nutritive and chemical attributes grown under two light conditions, harvested across three time points

Entry Number	Variety[a]	Company	Light sensitivity
E1	Autopilot 1.0	HiLo	Photoperiod-insensitive (autoflower)
E2	Blue Genius	Unique Botanicals	Photoperiod-insensitive (autoflower)
E3	NS-26	Phytonyx	Photoperiod-sensitive (full season)
E4	NS-52	Phytonyx	Photoperiod-sensitive (full season)
E5	Siskiyou OG	Phytonyx (distributed by Cascade Hemp Collective)	Photoperiod-sensitive (full season)

[a]All varieties are chemotype III.
